# Synthesis of Functionalized *N*-(4-Bromophenyl)furan-2-carboxamides via Suzuki-Miyaura Cross-Coupling: Anti-Bacterial Activities against Clinically Isolated Drug Resistant *A. baumannii*, *K. pneumoniae*, *E. cloacae* and MRSA and Its Validation via a Computational Approach

**DOI:** 10.3390/ph15070841

**Published:** 2022-07-08

**Authors:** Ayesha Siddiqa, Muhammad Zubair, Muhammad Bilal, Nasir Rasool, Muhammad Usman Qamar, Aqsa Khalid, Gulraiz Ahmad, Muhammad Imran, Sajid Mahmood, Ghulam Abbas Ashraf

**Affiliations:** 1 Department of Chemistry, Government College, University Faisalabad, Faisalabad 38000, Pakistan; ayeshasiddiqa096@gmail.com (A.S.); muhammadbilalgcuf@gmail.com (M.B.); nasirrasool@gcuf.edu.pk (N.R.); gulchemist35@gmail.com (G.A.); 2 Department of Microbiology, Faculty of Life Sciences, Government College, University Faisalabad, Faisalabad 38000, Pakistan; musmanqamar@gcuf.edu.pk; 3 School of Interdisciplinary Engineering & Science (SINES), National University of Sciences and Technology (NUST), Islamabad 44000, Pakistan; aqsa.khalid30@yahoo.com; 4 Department of Chemistry, Faculty of Science, King Khalid University, P.O. Box 9004, Abha 61413, Saudi Arabia; miahmad@kku.edu.sa; 5 Key Laboratory of the Ministry of Eduction for Advanced Catalysis Materials, Department of Chemistry, Zhejiang Normal University, Jinhua 312004, China; sajidmahmood1987@zjnu.edu.cn; 6 Department of Physics, Zhejiang Normal University, Jinhua 312004, China

**Keywords:** amide, cross-coupling, anti-bacterial, resistant, docking studies, MD simulations

## Abstract

*N*-(4-bromophenyl)furan-2-carboxamide (**3**) was synthesized by the reaction furan-2-carbonyl chloride (**1**) and 4-bromoaniline (**2**) in the presence of Et_3_N in excellent yields of 94%. The carboxamide (**3**) was arylated by employing triphenylphosphine palladium as a catalyst and K_3_PO_4_ as a base to afford *N*-(4-bromophenyl)furan-2-carboxamide analogues (**5a-i**) in moderate to good yields (43–83%). Furthermore, we investigated the in vitro anti-bacterial activities of the respective compounds against clinically isolated drug-resistant bacteria *A. baumannii*, *K. pneumoniae*, *E. cloacae* and *S. aureus*. The molecule (**3**) was found to be the most effective activity against these bacteria, particularly NDM-positive bacteria *A. baumannii* as compared to various commercially available drugs. Docking studies and MD simulations further validated it, expressing the active site and molecular interaction stability.

## 1. Introduction

The rise of widely drug-resistant (XDR) bacteria is a serious threat to public healthcare systems worldwide [1]. If anti-microbial resistant bacteria prevail, one life will be lost every three seconds in 2050, leading to an economic loss of more than $100 trillion [2]. Carbapenem-resistant *Klebsiella pneumonia* (CRKP), Carbapenem-resistant *Acinetobacter baumannii* (CRAB) and carbapenem-resistant *Enterobacter cloacae* (CREC) were placed on the “Critical Pathogens List” by the World Health Organization, while methicillin-resistant *Staphylococcus aureus* (MRSA) was placed on the “High Priority Pathogens List” by the WHO [3]. Bacteremia, septicemia, wound infections, skin, respiratory tract, urinary tract and nosocomial infections are all caused by these bacteria. These infections have developed resistance to a broad spectrum of antibiotics, including quinolones, β-lactams, and aminoglycosides, and can only be treated with toxigenic antibiotics like polymyxin and colistin [4]. The pathogens acquired resistance by various mechanisms such as an efflux pump in *A. baumannii* and *E. cloacae* [5,6], porin protein loss and decreased intake of antibiotics in *K. pneumoniae* [7], and production of PBP2a in *S. aureus* [8]. The development of new anti-microbial pharmaceuticals is still in demand since diseases and previously existing antibiotic agents have a diminishing effect on microbial resistance, which are significant risk factors for morbidity and mortality in both developed and underdeveloped countries [9,10,11]. *O*-heterocycles are found in a diverse range of pharmaceuticals, synthetic precursors, and natural products [12,13,14,15]. Carboxamide is an essential scaffold that can show antibacterial properties. The carboxamide bond (–CO–NH–) in proteins is indeed a pivotal building unit that has gotten a lot of attention due to its great hydrolysis resistance. This phenomenon is significant in biological systems because it allows peptides to be built from very simple amino acid substrates [16,17,18,19,20,21,22]. Moreover, Suzuki–Miyaura cross-coupling is an efficient tool that involves a pseudohalide/organohalide electrophile and organoboron nucleophile to establish a carbon–carbon bond in the presence of a catalyst [23,24].

Therefore, in the present work, *N*-(4-bromophenyl)furan-2-carboxamide (**3**) by the reaction of furan-2-carbonyl chloride (**1**) with 4-bromoaniline (**2**) in the presence of base trimethylamine (Et_3_N) and its derivatives (**5a-i**) were synthesized via Suzuki–Miyaura cross-coupling reactions [25]. The agar well diffusion method was used to evaluate all of the target compounds for antibacterial activity toward NDM-producing bacteria. Following that, the MBC and MIC were analyzed and validated by MD simulations and molecular docking studies.

## 2. Results and Discussion

### 2.1. Chemistry

**Scheme 1:**
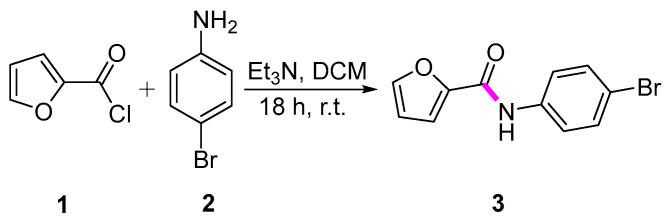
Synthesis of *N*-(4-bromophenyl)furan-2-carboxamide (**3**).

**Scheme 2:**
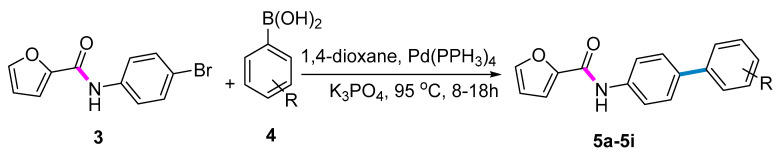
Synthesis of *N*-(4-arylphenyl)furan-2-carboxamide (**5a-i**).

**Figure 1:**
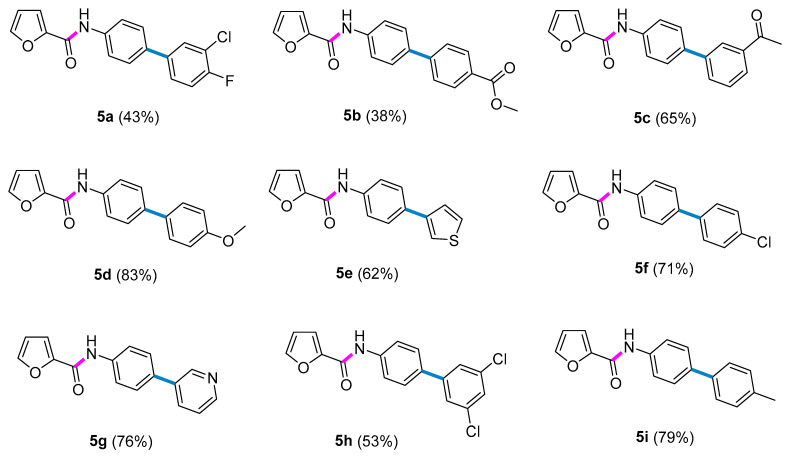
An overview of *N*-(4-aryl phenyl)furan-2-carboxamide (**5a-i**) via Suzuki–Miyamura cross-coupling reactions.

*N*-(4-bromophenyl)furan-2-carboxamide (**3**) was synthesized by the reaction of commercially available furan-2-carbonyl chloride (**1**) and 4-bromoaniline (**2**) in the presence of triethylamine base and dry dichloromethane (DCM) to afford the product in an excellent yield (94%) at room temperature (Scheme 1). Subsequently, the Suzuki–Miyamura cross-coupling reaction of *N*-(4-bromophenyl)furan-2-carboxamide (**3**) with various aryl and heteroaryl boronic acids (**4**) in the presence of the catalyst tetrakis(triphenylphosphine)palladium(0) and potassium phosphate as a base afforded the targeted compounds (**5a-i**) in fair to good yields (32–83%) (Scheme 2). The synthesized compounds were purified by flash column chromatography, and were characterized by spectroscopic techniques (^1^HNMR and ^13^CNMR), mass spectrometry, elemental analysis and their melting points, which are mentioned in Section 3.3. Our research group previously determined the effect of substituents on boronic acids, where the electron-donating groups were more responsible for good yields than the electron-withdrawing groups [26,27]. The same pattern can be observed in the synthesized compounds; the compound (**5b**) has the lowest yield (38%) due to the presence of electron-poor and bulky groups (Figure 1).

### 2.2. Antibacterial Activity

**Figure 2:**
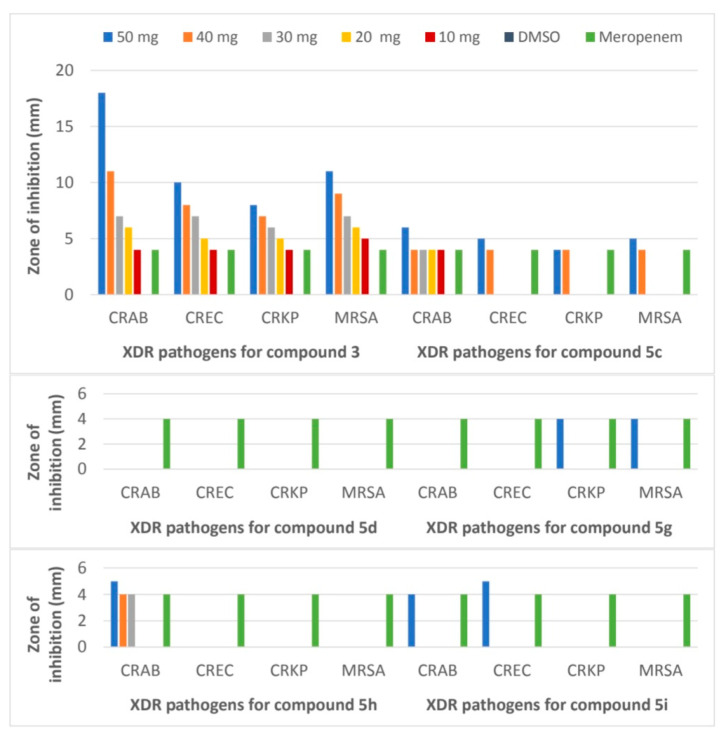
Agar well diffusion of different compounds against XDR pathogens.

The MIC values of various antibiotics exhibited in Table 1 show that Gram-negative bacterial strains were resistant to the AWaRe (Access, Watch, and Reserve) WHO classes of antibiotics such as β-lactams, aminoglycosides, and quinolones, except for polymyxin. Further, MRSA displayed resistance to β-lactams, and quinolones and was sensitive to vancomycin and linezolid. Phenotypic detection revealed that all the GNR were carbapenemase and MBL producers while Staphylococcus were MRSA. Therefore, in the present study, the molecules (**3**) and (**5a-i**) were screened for antibacterial activity at different concentrations (10, 20, 30 40, and 50 mg/well) against XDR pathogens (CRAB, CRKP, CREC, and MRSA) by the agar well diffusion method. MIC and MBC were calculated by broth dilution methods. The results presented in Table 1 show that only compound (**3**) exhibited excellent activity against XDR pathogens. However, compound (**5c**) displayed low activity as compared to compound (**3**), while the other molecules failed to present good activity. The zone of inhibition (mm) increased against all XDR pathogens with the increase in compound concentration. Compound (**3**) at 50 mg concentration showed the highest zone of inhibition (18 mm) against CRAB as compared to other pathogens while meropenem antibiotic was inhibited at a 4 mm zone of inhibition (Figure 2). The values of MBC and MIC of compound (**3**) were calculated against all tested XDR pathogens and the results showed that CRAB, CREC, and CRKP exhibited a MIC of 6.25 mg and MBC of 12.5 mg respectively, while MRSA exhibited a MIC of 12.5 mg and MBC of 25 mg, respectively (Table 2).

### 2.3. Molecular Docking Study

#### Molecular Docking Study of the *NDM-1 A. baumannii*/**3**, **5a-i** Compounds

**Figure 3:**
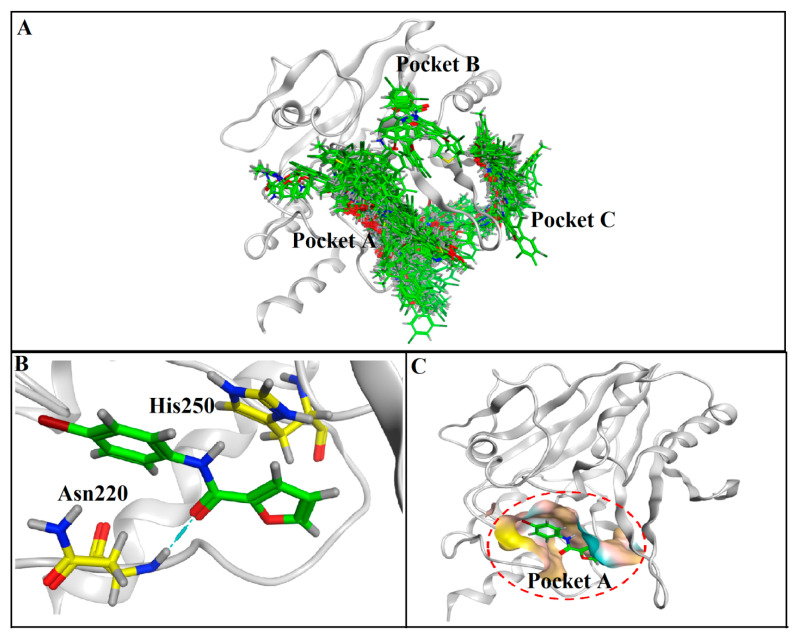
Conformational analysis and interaction pattern of NDM-1-embelin protein with highly active compound **3**. (**A**) Three major binding pockets (pocket A, pocket B and Pocket C) were identified and the majority of the ligand conformations found in the pocket A of NDM-1 protein, which enhances the confidence that pocket A is the most probable binding cavity. (**B**) Molecular interactions (hydrogen bonding and hydrophobic) of highly active compound **3** with NDM-1 protein. (**C**) The least energy conformation of compound **3** residing in pocket A, depicting the significant role of Pocket A in ligand binding.

The broad conformation zone of (**3**, **5a-i**) compounds surrounded by the NDM-1 active site was investigated using molecular docking. The active site of NDM-1 was overlaid with the 100 binding conformations for each drug (**3**, **5a-i**). The superposition of all the conformations of ligands (**3**, **5a-i**) displays three major binding pockets (Pocket A, Pocket B and Pocket C) as shown in Figure 3A. Interestingly, the final selected confirmation based on the highest Gold score and least energy value showed the ligands binding in the pocket A (Figures S27 and S28). Moreover, the docked complexes represent a similar binding pattern (Figure 3A) with distinct binding interactions (Figure 3B) towards NDM-1 protein. These results indicate that pocket A is the most probable cavity for the inhibition of NDM-1-embelin protein (Figure S27). Moreover, the non-bonded interactions of compound (**3**) at the NDM-1-embelin interface were determined to describe the important interacting residues that could be incorporated in the design of potent inhibitors with improved biological activity. Almost all of the compounds (**3**, **5a-i**) were embedded in the NDM-1 active site (Pocket A), demonstrating that NDM-1 and ligands had a broad van der Waals interaction (**3**, **5a-i**) (Supplementary Information Figure S27). The catalytic corner of the enzyme binds to the N–H of amide functionality of (**3**, **5a-i**) compounds, whereas the other part points out of this corner. Compound (**3**) displayed hydrogen bonding (OH–NH) between the carbonyl group of the amide substitution and Asn220 amino acid residue. Moreover, the hydrophobic interactions, Pi–H and Pi–Pi, were also observed with Asn220 and His250 amino acid residues. The compound (**5c**) displayed only hydrophobic (Pi–H) interactions with His120 and Asn220. Similarly, the compounds (**5g**, **5h** and **5i**) also displayed hydrophobic interactions with His120, Asn220, Lys216, and His250. The activities of these compounds were predicted experimentally, and the parent compound (**3**) and its derivative (**5c**) showed high biological activity in comparison to others. This might be because compound (**3**) showed both hydrogen bonding and hydrophobic interactions, which are missing in the rest of the dataset. The importance of the coordination bond in collaboration and recognition of the NDM-1-ligand with a viable drug was established in a prior docking study of embelin with NDM-1, emphasizing the importance of the coordination bond in collaboration and detection of NDM-1-ligand with a suitable drug. It was proposed that compound (**3**) regained meropenem activity towards a panel of NDM-positive pathogens, including *K. pneumoniae*, *A. baumannii*, and *E. coli*, due to the strong hydrogen bonding and hydrophobic interaction between (**3**, **5a-i**) and NDM-1 [28]. The antibacterial activities against NDM-1 *A. baumannii* were confirmed by a docking analysis of corresponding furan derivatives, which revealed that compounds (**3** and **5c**) had the best meropenem activity towards NDM-positive bacteria, with comparable results to our compounds (**5g**, **5h**, and **5i**). Compound (**3**) was discovered to be a strong antibacterial antibiotic hybrid against NDM-1 *A-baumannii* in a preliminary study (Figure 3). As a result, we predicted that (**3**) and its derivative (**5c**) could be effective carbapenem adjuvants for bacteria that produce NDM-1. To validate our hypothesis, the MD simulation of the experimentally tested compounds (**3**, **5c**, **5g**, **5h**, and **5i**) was performed using the GROMACS (Supplementary Information Figures S29–S33).

**Figure 4:**
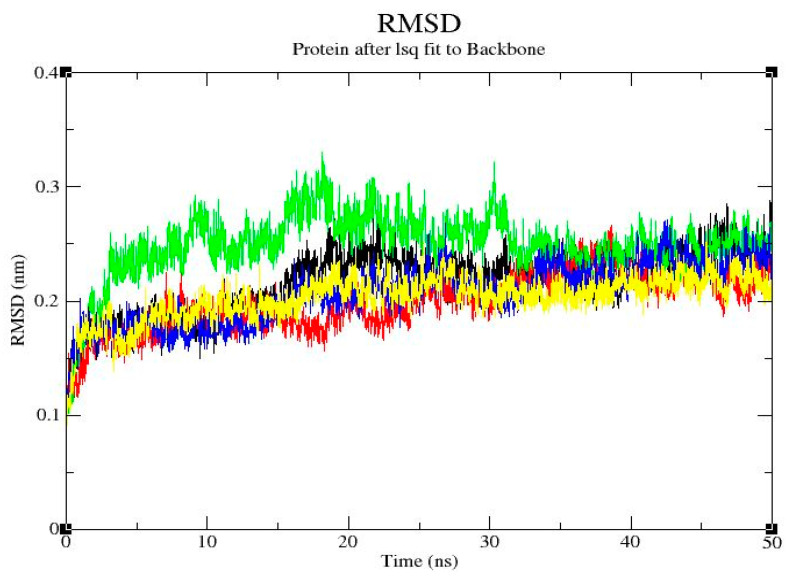
Molecular Dynamics (MD) simulation of NDM-1 with compound (**3**) and its structural analogues (**5c**, **5g-i**) over 50 ns. RMSD trajectory of NDM1-**3** complex (black color). RMSD trajectory of NDM1-**5c** complex (red color). RMSD trajectory of NDM1-**5g** complex (blue color). RMSD trajectory of NDM1-**5h** complex (green color). RMSD trajectory of NDM1-**5i** complex (yellow color).

The RMSD graph obtained between the protein backbone and the ligands throughout the 50 ns MD simulation ensures the stability of the protein–ligand complexes. It has been observed (Figure 4) that the RMSD plots of *NDM-1 A. baumannii*/(**3**, **5c**, and **5g-i**) compounds showed that all the complexes achieve stability after 20 ns of the simulation. This may infer the high binding affinities and better binding fit of the ligands within the active site of the 4EXS receptor. The overall RMSD analysis implicated that compound (**3**) and its structural analogues (**5c**, **5g-i**) are important for the inhibition of NDM-1 protein (individual RMSD plots given in Supplementary Information Figures S29–S33).

## 3. Materials and Methods

### 3.1. General Information

All the chemicals were sourced from Sigma-Aldrich and Alfa-Aesar. The moisture and air-sensitive reaction were done in an inert environment. The compounds or reaction mixture were dried by a rotary evaporator. The reaction progress was checked by thin-layer chromatography (silica gel 60 PF254 cards) and the reaction mixture was separated through column chromatography by using Silica gel (230–400 mesh). The synthesized compounds were confirmed by spectroscopic analysis, a 500 MHz Bruker NMR spectrometer in the presence of solvent deuterated dimethyl sulfoxide (DMSO-d6). Mass spectra were recorded on a JEOL spectrometer (JMS-HX-110, USA).

### 3.2. General Procedure for the Synthesis of Compounds

#### 3.2.1. Synthesis of *N*-(4-Bromophenyl)furan-2-carboxamide (**3**)

In a clean and dried schlenk flask, 4-bromo aniline (1.0 eq., 2.30 mmol) was added in a 15 mL dry dichloromethane (DCM) solvent. Subsequently, triethylamine (1.0 eq., 2.30 mmol) was poured into the reaction mixture and it was placed in an ice bath to chill it down to 0 °C. Then, furan-2-carbonyl chloride (1.0 eq., 2.30 mmol) was added to the reaction mixture at room temperature (r.t.) for 18 h. TLC was used to monitor the reaction completion. After that, a 1N HCl solution was added to the dried reaction mixture, and DCM was used to quench the product from the aqueous layer. After the separation of the organic layer, the saturated sodium bicarbonate (NaHCO_3_) solution and brine were added. The DCM layer was separated and dried after vigorous shaking. The components of the reaction mixture were separated by flash column chromatography. Spectroscopic examination confirmed the final product [29].

#### 3.2.2. Arylation of *N*-(4-Bromophenyl)furan-2-carboxamide (**5a-i**)

At room temperature, *N*-(4-bromophenyl)furan-2-carboxamide (1.0 eq., 0.488 mmol) was poured into a Schlink tube in 5 mL of 1,4-dioxane under inert argon atmosphere, along with the tetrakis(triphenylphosphine)palladium(0) catalyst, and agitated for 30 min. Then, aryl or heteroaryl boronic acids (1.1 eq., 0.537 mmol), K_3_PO_4_ (1.0 eq., 0.488 mmol) as a base, and 0.5 mL water were added. The reaction was then set to reflux for 8–18 h. TLC was used to examine the reaction’s progress. Following the completion of the reaction, the reaction mixture was washed with ethyl acetate and dried using a rotary evaporator. The required product was then purified by using flash column chromatography with ethyl acetate and n-hexane (20:80). NMR spectroscopy and mass spectroscopy analysis was used to characterize the desired product [30].

### 3.3. Characterization Data

#### 3.3.1. *N*-(4-Bromophenyl)furan-2-carboxamide (**3**)

White solid, M.P = 257–260 °C; ^1^H NMR (500 MHz, DMSO) δ 10.31 (s, 1H), 7.94 (dd, *J* = 1.6, 0.6 Hz, 1H), 7.79–7.68 (m, 2H), 7.52 (d, *J* = 8.9 Hz, 2H), 7.33 (dd, *J* = 3.5, 0.6 Hz, 1H), 6.70 (dd, *J* = 3.5, 1.7 Hz, 1H). ^13^C NMR (126 MHz, DMSO) δ 156.71, 147.72, 146.38, 138.41, 138.16, 131.94, 122.70, 115.55, 112.71. EI/MS *m*/*z* (%): 267.2 [M+H]^+^; [M-Cl] = 186.1. Analytically calculated for C_11_H_8_BrNO_2_: C, 49.65; H, 3.03; N, 5.26. Found: C, 49.71; H, 3.06; N, 5.21.

#### 3.3.2. *N*-(3′-Chloro-4′-fluorobiphenyl-4-yl)furan-2-carboxamide (**5a**)

Light Brown solid, M.P = 312–318 °C; ^1^H NMR (500 MHz, DMSO) δ 10.25 (s, 1H), 7.95 (d, *J* = 1.0 Hz, 1H), 7.82 (dd, *J* = 8.1, 5.0 Hz, 3H), 7.71 (d, *J* = 8.7 Hz, 2H), 7.64 (dd, *J* = 5.0, 2.9 Hz, 1H), 7.56 (dd, *J* = 5.0, 1.2 Hz, 1H), 7.35 (d, *J* = 3.4 Hz, 1H), 6.72 (dd, *J* = 3.4, 1.7 Hz, 1H). ^13^C NMR (126 MHz, DMSO) δ 156.62, 147.98, 146.21, 141.57, 138.04, 131.13, 127.46, 126.76, 126.51, 121.04, 120.56, 115.24, 112.66. EI/MS *m/z* (%): 316.8 [M+H]^+^; [M-Cl] = 280.14, [M-F, Cl] = 261.3. Analytically calculated for C_17_H_11_ClFNO_2_: C, 64.67; H, 3.51; N, 4.44. Found: C, 64.72; H, 3.53; N, 4.41.

#### 3.3.3. Methyl 4′-(Furan-2-carboxamido)biphenyl-4-carboxylate (**5b**)

Off-white solid, M.P = 360–364 °C; ^1^H NMR (500 MHz, DMSO) δ 10.35 (s, 1H), 8.03 (d, *J* = 8.3 Hz, 2H), 7.97 (s, 1H), 7.91 (d, *J* = 8.7 Hz, 2H), 7.85 (d, *J* = 8.3 Hz, 2H), 7.77 (d, *J* = 8.7 Hz, 2H), 7.38 (d, *J* = 3.5 Hz, 1H), 6.73 (dd, *J* = 3.4, 1.7 Hz, 1H), 3.88 (s, 3H). ^13^C NMR (126 MHz, DMSO) δ 167.34, 162.12, 146.34, 134.51, 130.29, 129.37, 127.73, 127.36, 126.92, 122.39, 121.08, 119.94, 115.45, 52.61. EI/MS *m/z* (%): 322.4 [M+H]^+^; [M-COOCH_3_] = 262.4. Analytically calculated for C_19_H_15_NO_4_: C, 71.02; H, 4.71; N, 4.36. Found: C, 71.10; H, 4.72; N, 4.32.

#### 3.3.4. *N*-(3′-Acetylbiphenyl-4-yl)furan-2-carboxamide (**5c**)

White solid, M.P = 373–375 °C; ^1^H NMR (500 MHz, DMSO) δ 10.34 (s, 1H), 8.20 (s, 1H), 7.98–7.89 (m, 4H), 7.76 (d, *J* = 8.6 Hz, 2H), 7.62 (t, *J* = 7.7 Hz, 1H), 7.37 (d, *J* = 3.5 Hz, 1H), 6.74–6.72 (m, 1H), 2.67 (s, 3H). EI/MS *m/z* (%): 306.5 [M+H]^+^; [M-COCH_3_] = 262.4. Analytically calculated for C_19_H_15_NO_3_: C, 74.74; H, 4.95; N, 4.59. Found: C, 74.80; H, 4.97; N, 4.57.

#### 3.3.5. *N*-(4′-Methoxybiphenyl-4-yl)furan-2-carboxamide (**5d**)

White solid, M.P = 335–338 °C; ^1^H NMR (500 MHz, DMSO) δ 10.26 (s, 1H), 8.00–7.92 (m, 1H), 7.83 (d, *J* = 8.7 Hz, 2H), 7.61 (d, *J* = 8.7 Hz, 4H), 7.36 (d, *J* = 3.5 Hz, 1H), 7.01 (d, *J* = 8.7 Hz, 2H), 6.72 (dd, *J* = 3.4, 1.7 Hz, 1H), 3.79 (s, 3H). ^13^C NMR (126 MHz, DMSO) δ 159.12, 156.63, 148.01, 146.18, 137.83, 135.62, 132.56, 127.85, 126.74, 121.15, 115.21, 114.80, 112.65, 55.62. EI/MS *m/z* (%): 294.5 [M+H]^+^; [M-OCH_3_] = 262.4. Analytically calculated for C_18_H_15_NO_3_: C, 73.71; H, 5.15; N, 4.78. Found: C, 73.77; H, 5.17; N, 4.77.

#### 3.3.6. *N*-(4-(Thiophen-3-yl)phenyl)furan-2-carboxamide (**5e**)

Off white solid, M.P = 352–354 °C; ^1^H NMR (500 MHz, DMSO) δ 10.31 (s, 1H), 7.97 (s, 1H), 7.92–7.84 (m, 3H), 7.69 (dd, *J* = 8.7, 5.6 Hz, 3H), 7.50 (t, *J* = 9.0 Hz, 1H), 7.37 (d, *J* = 3.5 Hz, 1H), 6.73 (dd, *J* = 3.4, 1.7 Hz, 1H). ^13^C NMR (126 MHz, DMSO) δ 156.97, 146.31, 138.97, 133.88, 128.64, 127.74, 127.50, 124.02, 121.06, 119.24, 117.85, 115.41, 112.73. EI/MS *m/z* (%): 270.4 [M+H]^+^; 271.4 [M+2]. Analytically calculated for C_15_H_11_NO_2_S: C, 66.89; H, 4.12; N, 5.20. Found: C, 66.95; H, 4.13; N, 5.18.

#### 3.3.7. *N*-(4′-Chlorobiphenyl-4-yl)furan-2-carboxamide (**5f**)

Off white solid, M.P = 324–327 °C; ^1^H NMR (500 MHz, DMSO) δ 10.28 (s, 1H), 7.96 (d, *J* = 0.9 Hz, 1H), 7.85 (d, *J* = 8.7 Hz, 2H), 7.64 (dt, *J* = 17.6, 8.9 Hz, 4H), 7.35 (dd, *J* = 8.5, 6.3 Hz, 2H), 7.21 (d, *J* = 8.3 Hz, 1H), 6.73 (dd, *J* = 3.5, 1.7 Hz, 1H). EI/MS *m/z* (%): 298.9 [M+H]^+^; [M-Cl] = 262.4. Analytically calculated for C_17_H_12_ClNO_2_: C, 68.58; H, 4.06; N, 4.70. Found: C, 68.61; H, 4.10; N, 4.68.

#### 3.3.8. *N*-(4-(Pyridin-3-yl)phenyl)furan-2-carboxamide (**5g**)

Light brown solid, M.P = 344–347 °C; ^1^H NMR (500 MHz, DMSO) δ 10.26 (s, 1H), 7.96 (d, *J* = 0.8 Hz, 1H), 7.84 (d, *J* = 8.6 Hz, 1H), 7.74 (d, *J* = 8.8 Hz, 2H), 7.63 (d, *J* = 8.6 Hz, 1H), 7.53 (d, *J* = 8.8 Hz, 2H), 7.36 (dd, *J* = 5.5, 3.5 Hz, 2H), 7.27 (s, 1H), 6.72 (dd, *J* = 4.7, 3.0 Hz, 1H). ^13^C NMR (126 MHz, DMSO) δ 156.71, 147.95, 146.37, 138.32, 131.94, 129.00, 127.24, 124.62, 122.71, 121.06, 115.55, 112.71. EI/MS *m/z* (%): 265.5 [M+H]^+^; 266.5 [M+2]. Analytically calculated for C_16_H_12_N_2_O_2_: C, 72.72; H, 4.58; N, 10.60. Found: C, 72.76; H, 4.61; N, 10.57.

#### 3.3.9. *N*-(3′,5′-Dichlorobiphenyl-4-yl)furan-2-carboxamide (**5h**)

Off white solid, M.P = 366–369 °C; ^1^H NMR (500 MHz, DMSO) δ 10.35 (s, 1H), 7.98–7.88 (m, 2H), 7.76 (dd, *J* = 12.4, 8.8 Hz, 2H), 7.63 (dd, *J* = 11.2, 7.7 Hz, 2H), 7.58–7.54 (m, 1H), 7.46 (d, *J* = 7.2 Hz, 1H), 7.36 (dd, *J* = 11.9, 3.5 Hz, 1H), 6.78–6.70 (m, 1H). ^13^C NMR (126 MHz, DMSO) δ 156.74, 147.86, 146.35, 139.65, 131.99, 129.28, 129.19, 127.64, 122.68, 120.94, 115.50, 112.72, 109.82. EI/MS *m/z* (%): 333.3 [M+H]^+^; [M-2Cl] = 261.3. Analytically calculated for C_17_H_11_C_l2_NO_2_: C, 61.47; H, 3.34; N, 4.22. Found: C, 61.51; H, 3.37; N, 4.19.

#### 3.3.10. *N*-(4′-Methylbiphenyl-4-yl)furan-2-carboxamide (**5i**)

White solid, M.P = 303–305 °C; ^1^H NMR (500 MHz, DMSO) δ 10.28 (s, 1H), 7.97–7.94 (m, 1H), 7.84 (d, *J* = 8.6 Hz, 2H), 7.64 (d, *J* = 8.6 Hz, 2H), 7.57 (d, *J* = 7.9 Hz, 2H), 7.36 (d, *J* = 3.5 Hz, 1H), 7.26 (d, *J* = 8.2 Hz, 2H), 6.72 (dd, *J* = 3.4, 1.7 Hz, 1H), 2.34 (s, 3H). EI/MS *m/z* (%): 278.5 [M+H]^+^; [M-CH_3_] = 262.4. Analytically calculated for C_18_H_15_NO_2_: C, 77.96; H, 5.45; N, 5.05. Found: C, 78.03; H, 5.51; N, 5.01.

### 3.4. Anti-Bacterial Activity

#### 3.4.1. Identification of the Bacterial Strains

*K. pneumoniae*, *A. baumannii*, *E. cloacae* and *S. aureus* were isolated from blood samples using BACTEC/Alert (Marcy-l’Étoile, France). The isolates were sub-cultured on MacConkey agar, blood and UTI chrome agar (Aldrich Sigma, St. Louis, MO, USA) and confirmed by VITEK 2^®^ compact system (Biomerieux, Marcy-l’Étoile, France).

#### 3.4.2. Antibiogram of the Isolates

The VITEK 2^®^ compact system was used to determine the MIC (µg/mL) of different antibiotics for these infections (BioMerieux, Marcy-l’Étoile, France). Ticarcillin/clavulanic acid, penicillin, cefuroxime, cefoxitin, ampicillin/sulbactam, cefixime, piperacillin, ceftriaxone, aztreonam, cefepime, meropenem, moxifloxacin, levofloxacin, tigecycline, minocycline, tetracycline, colistin and trimethoprim CLSI guidelines 2020 were used to evaluate the susceptibility (Table 3).

#### 3.4.3. Phenotypic Determination of MRSA

MRSA was evaluated by the agar disc diffusion method, as defined by the CLSI 2020. MRSA was dispersed on a Mueller Hinton agar (MHA) plate, and a cefoxitin (30 μg) disc was placed on top of it. MRSA was declared positive if the zone of inhibition was less than 21 mm [31].

#### 3.4.4. Phenotypic Detection of Carbapenemase Enzyme

**Figure 5:**
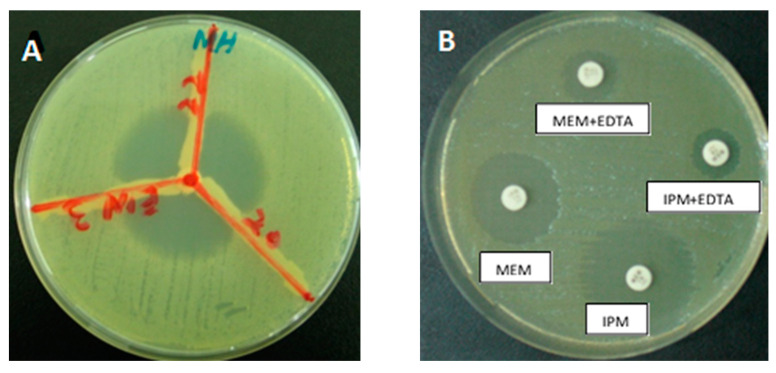
(**A**) Modified Hodge’s test for carbapenemase; (**B**) Double disc synergy method.

The Modified Hodge’s test was used for the carbapenemase activity to test the Gram-negative isolates [32]. The isolates were loaded onto the MHA plate, the meropenem disc was inserted in the center, and test organisms were streaked from the disc’s edge to the plates’ edge. A cloverleaf-like indentation indicated the existence of carbapenemase enzymes after overnight incubation (Figure 5A).

#### 3.4.5. Phenotypic Detection of Metallo-β-Lactamase (MBL) Enzyme

MBL-producing bacteria were identified by utilizing a double disc synergistic method with EDTA, as previously described. In a nutshell, the isolates were placed out on MHA plates with two imipenem and meropenem discs spaced 24 mm apart. Each carbapenem disc received 10 ul of 0.5 M EDTA solution (Figure 5B). If the EDTA discs had a greater inhibition zone of >5 mm than the non-EDTA discs, the test was declared positive [33].

### 3.5. Antibacterial Activity of the Compound against XDR Pathogens

#### 3.5.1. Agar Well Diffusion Method

The antibacterial activity of the compounds towards XDR *E. coli* was tested using an agar well diffusion method. After injecting a 0.5 McFarland bacterial suspension on the Mueller Hinton Agar plate, a cleaned 6 mm cork borer was used to create wells on each plate. The plates were incubated overnight at 37 °C when the drugs (10 mg, 20 mg, 30 mg, 40 mg, and 50 mg) were poured into the wells and diluted in 100 μL DMSO. A Vernier caliper was used to determine the Zone of Inhibition (mm). The experiment was carried out three times. A meropenem (10 g) disc was used as an antibiotic control (Figure 2, Table 1) [34].

#### 3.5.2. Minimum Inhibitory Concertation of Different Compounds against XDR Pathogens

The MIC (% *w*/*v*) of different compounds was determined by the micro broth dilution method. Two to three different colonies were mixed in double-strength lysogeny broth (LB) media (20 mL) and cultured overnight at 37 °C in a Falcon 50 mL tube. The bacterial suspension was diluted to 0.5 McFarland at an optical density (OD) of 0.07 at 600 nm. In a nutshell, each molecule was serially diluted in DMSO (0.76, 1.56, 3.12, 6.25, 12.5, 25, 50 mg), and 100 μL of each dilution was applied to 96-well flat-bottom microtiter plates (Thermo Fisher Scientific, Leicestershire, UK). A 100 μL bacterial suspension was then poured into each well. The positive-control wells had LB with bacterial suspension, while the negative-control wells had 100 μL of LB. In a shaking incubator (Thermo Fisher Scientific’s MaxQTM Mini 4450), the microtiter plate was shaken at 3 g overnight at 37 °C. The MIC was calculated by comparing each well to positive and negative control wells. All of the operations were repeated three times (Table 2) [34].

#### 3.5.3. Determination of MBC

The initial dilution with no growth on the agar plate is characterized as the minimum bactericidal concentration (MBC, % *w*/*v*). A sample of 10 μL was collected from a microtiter plate with no visible growth wells, injected on nutrient agar plates (Oxoid, Hampshire, UK), and aerobically cultured for 24 h at 37 °C. The viability of the cells on the plates was checked, and any colonies that formed were rated as either bacterial growth or no bacterial growth. All procedures were carried out three times (Table 2) [34].

### 3.6. Docking Studies

Gold software was used to examine the interaction of intermolecular *N*-(4-bromophenyl)furan-2-carboxamide (**3**) and its analogues (**5a-i**) with NDM-positive *A. baumannii* protein [35]. The Gold score fitness function was used to estimate the binding energies of a produced ligand with a protein receptor. One hundred conformations of the NDM-1 active site per ligand were predicted to eliminate any basis in the docking process. A high-resolution crystal structure of NDM-1 bonded to L-captopril (PDB ID: 4EXS) from the Protein Data Bank (PDB) was chosen as a model for the entire docking investigation [36]. The binding site was sampled by keeping a distance of 20 Å around the amino acid residues Asn220, Cys208, His250, Gly219, His122, Asp124, His189, Val73, His120, Met67, and Trp93 [36]. These previously known amino acid residues from mutagenesis data are implicated to be crucial in defining the binding site/region of NDM-1 protein for the docking study of *N*-(4-bromophenyl)furan-2-carboxamide (**3**) derivatives. The X-ray crystallographic structure of NDM-1(4EXS) was prepared before the docking by the removal of L-captopril and water molecules. Moreover, the 4EXS was optimized by the protonation at a pH of 7.0 followed by the energy minimization using Amber99 force field in the Molecular Operating Environment (MOE). Furthermore, the *N*-(4-bromophenyl)furan-2-carboxamide (**3**) and its analogues (**5a-i**) were also energy minimized using the MMFF9x force field in MOE. Both the 4EXS structure and ligands were subjected to molecular docking for protein–ligand interaction analysis [37].

### 3.7. Molecular Dynamics (MD) Simulation

Molecular dynamics (MD) simulation was performed using the CHARMM36 force field in the GROMACS software [38]. The ligand (**3**, **5c**, **5g**, **5h**, and **5i**) topology files compatible with the receptor (4EXS) force field (CHARMM36) were obtained from the CGenFF server. The system was solvated using the TIP3P water model to represent the explicit water molecules. Moreover, the Na^+^ ions were also added to neutralize the system. The subsequent NVT and NPT equilibration simulations were carried out at 300 K using the Nosé–Hoover thermostat and the Parrinello–Rahman barostat, respectively [39]. After the energy minimization and equilibration steps, the MD simulation was performed for all the experimentally tested compounds to check their stability in the binding pocket over 50 ns. The root-mean-square deviation (RMSD) graphs of the simulated NDM-1 *A. baumannii* (**3**) and its analogues (**5c**, **5g**, **5h**, **5i**) were plotted through the QTgrace software to analyze the stability of complexes (**4EXS-3**, **5c**, **5g-5i**) over 50 ns of MD simulation.

## 4. Conclusions

We synthesized *N*-(4-bromophenyl)furan-2-carboxamide (**3**) with an excellent yield (94%). A series of functionalized molecules (**5a-i**) were synthesized by the Suzuki–Miyaura cross-coupling reaction in moderate to good yields (43–83%). The molecule (**3**) was found to be the most effective compound against these bacteria, particularly NDM-positive *A. baumannii* as compared to various commercially available drugs. Docking studies and MD simulations revealed the stability of the protein–ligand complexes. All of the compounds (**3**, **5a-i**) were embedded into the NDM-1 active site but compound (**3**) displayed hydrogen bonding and hydrophobic interactions: Pi-H and Pi-Pi. Further validation showed the stability of the protein–ligand complexes. The compound (**3**) was the most valuable and proved to be a potential antibacterial agent.

## Figures and Tables

**Table 1:** Agar well diffusion of different compounds against XDR pathogens.

Comp. No.	Bacterial Strains	Zone (mm) (50 mg)	Zone (mm) (40 mg)	Zone (mm) (30 mg)	Zone (mm) (20 mg)	Zone (mm) (10 mg)	Zone (mm) DMSO	Zone (mm) Meropenem
**3**	CRAB	18 ± 1	11 ± 2	7 ± 2	6 ± 2	4 ± 0	0 ± 0	4
	CREC	10 ± 2	8 ± 3	7 ± 3	5 ± 2	4 ± 0	0 ± 0	4
	CRKP	8 ± 1	7 ± 2	6 ± 2	5 ± 2	4 ± 0	0 ± 0	4
	MRSA	11 ± 2	9 ± 1	7 ± 2	6 ± 2	5 ± 0	0 ± 0	4
**5c**	CRAB	6 ± 3	4 ± 2	4 ± 2	4 ± 3	4 ± 0	0 ± 0	4
	CREC	5 ± 2	4 ± 1	0 ± 0	0 ± 0	0 ± 0	0 ± 0	4
	CRKP	4 ± 1	4 ± 2	0 ± 0	0 ± 0	0 ± 0	0 ± 0	4
	MRSA	5 ± 1	4 ± 2	0 ± 0	0 ± 0	0 ± 0	0 ± 0	4
**5d**	CRAB	0 ± 0	0 ± 0	0 ± 0	0 ± 0	0 ± 0	0 ± 0	4
	CREC	0 ± 0	0 ± 0	0 ± 0	0 ± 0	0 ± 0	0 ± 0	4
	CRKP	0 ± 0	0 ± 0	0 ± 0	0 ± 0	0 ± 0	0 ± 0	4
	MRSA	0 ± 0	0 ± 0	0 ± 0	0 ± 0	0 ± 0	0 ± 0	4
**5g**	CRAB	0 ± 0	0 ± 0	0 ± 0	0 ± 0	0 ± 0	0 ± 0	4
	CREC	0 ± 0	0 ± 0	0 ± 0	0 ± 0	0 ± 0	0 ± 0	4
	CRKP	4 ± 2	0 ± 0	0 ± 0	0 ± 0	0 ± 0	0 ± 0	4
	MRSA	4 ± 3	0 ± 0	0 ± 0	0 ± 0	0 ± 0	0 ± 0	4
**5h**	CRAB	5 ± 2	4 ± 2	4 ± 2	0 ± 0	0 ± 0	0 ± 0	4
	CREC	0 ± 0	0 ± 0	0 ± 0	0 ± 0	0 ± 0	0 ± 0	4
	CRKP	0 ± 0	0 ± 0	0 ± 0	0 ± 0	0 ± 0	0 ± 0	4
	MRSA	0 ± 0	0 ± 0	0 ± 0	0 ± 0	0 ± 0	0 ± 0	4
**5i**	CRAB	4 ± 2	0 ± 0	0 ± 0	0 ± 0	0 ± 0	0 ± 0	4
	CREC	5 ± 1	0 ± 0	0 ± 0	0 ± 0	0 ± 0	0 ± 0	4
	CRKP	0 ± 0	0 ± 0	0 ± 0	0 ± 0	0 ± 0	0 ± 0	4
	MRSA	0 ± 0	0 ± 0	0 ± 0	0 ± 0	0 ± 0	0 ± 0	4

**Table 2:** MIC (mg/)mL and MBC of compound (**3**) against XDR pathogens.

**Pathogens**	**MIC**	**MBC**
CRAB	6.25	12.5
CREC	6.25	12.5
CRKP	6.25	12.5
MRSA	12.5	25

**Table 3:** MIC (µg/mL) of antibiotics against XDR pathogens.

Antibiotics	MIC Breakpoints (µg/mL)	*A. baumannii*	*K. pneumoniae*	*E. cloacae*	*S. aureus*
P	≤0.12–≥0.25	NA	NA	NA	≥16
E	≤0.5–≥8	NA	NA	NA	≥32
DA	≤0.5–≥4	NA	NA	NA	≥16
SAM	≤8/4–≥32/16	≥64/128	≥64/32	≥64/128	NA
PIP	≤16–≥128	≥256	≥256	≥256	NA
FOX	≤4–≥8	NA	NA	NA	≥32
CRO	≤8–≥64	≥256	≥256	≥128	≥128
FEP	≤8–≥32	≥256	≥128	≥128	≥64
ATM	≤4–≥16	≥64	≥64	≥64	≥64
MEM	≤2–≥8	≥32	≥32	≥32	≥8
LEV	≤1–≥4	≥16	≥16	≥16	0.5
MXF	≤0.5–≥2	≥16	≥16	≥16	NA
MNO	≤4–≥16	≥64	≥64	≥32	NA
TE	≤4–≥16	≥32	≥32	≥32	NA
C	≤8–≥32	≥64	≥128	≥64	NA
SXT	≤2/38–≥4/76	≥8/304	≥8/304	≥8/304	≥8/152
VA	≤2–≥16	NA	NA	NA	1
LZD	≤4–≥8	NA	NA	NA	2
CS	≥4	0.5	0.5	0.5	NA

P: Penicillin; DA: Clindamycin; E: Erythromycin; SAM: Ampicillin/sulbactam, CRO: Ceftriaxone; PIP: Piperacillin; ATM: Aztreonam; FOX: Cefoxitin; MEM: Meropenem; FEP: Cefepime; MNO: Minocycline; MXF: Moxifloxacin; LEV: Levofloxacin; TE: Tetracycline; C: Chloramphenicol; SXT: Co-trimethoprim; TGC: Tigecycline; VA: Vancomycin; CS: Colistin, LZD: Linezolid, NA: Not applicable.

## Data Availability

Data is contained within the article and Supplementary Materials.

## Supplementary Materials

The following supporting information can be downloaded at: https://www.mdpi.com/article/10.3390/ph15070841/s1.
