# Supplementary material for: Synthesis of Functionalized N-(4-Bromophenyl)furan-2-carboxamides via Suzuki-Miyaura Cross-Coupling: Anti-Bacterial Activities against Clinically Isolated Drug Resistant A. baumannii, K. pneumoniae, E. cloacae and MRSA and Its Validation via a Computational Approach
Source: Pharmaceuticals (Basel). 2022 Jul 8;15(7):841. doi: 10.3390/ph15070841 (PMC9319355; doi:10.3390/ph15070841)
Supplement: Supplementary file 1 [file pharmaceuticals-15-00841-s001.zip › pharmaceuticals-1749915-supplementary.pdf]

N-(4-bromophenyl)furan-2-carboxamides via Suzuki-miyaura Cross-Coupling: Anti-bacterial activities against clinically isolated drug resistant *A. baumannii, K. pneumoniae, E. cloacae and* MRSA and its validation via computational approach.

Ayesha Siddiqa [1], Muhammad Zubair [1,*], Muhammad Bilal [1], Nasir Rasool [1], Muhammad Usman Qamar [2], Aqsa khalid [3], Gulraiz Ahmad [1], Muhammad Imran [4], Sajid Mahmood [5], Ghulam Abbas Ashraf [6,*]

[1] Department of Chemistry, Government College, University Faisalabad, 38000, Pakistan; ayeshasiddiqa096@gmail.com (A.S.), zubairmkn@gcuf.edu.pk (M.Z.), muhammadbilalgcuf@gmail.com (M.B.), nasirrasool@gcuf.edu.pk (N.R.), gulchemist35@gmail.com (G.A.)

[2] Department of Microbiology, Faculty of Life Sciences, Government College, University Faisalabad, 38000, Pakistan; musmanqamar@gcuf.edu.pk (M.U.Q.)

[3] School of Interdisciplinary Engineering & Science (SINES), National University of Sciences and Technology (NUST), Islamabad 44000, Pakistan; aqsa.khalid30@yahoo.com (A.K.)

[4] Department of Chemistry, Faculty of Science, King Khalid University, P.O. Box 9004, Abha 61413, Saudi Arabia; miahmad@kku.edu.sa (M.I.))

[5] Key Laboratory of the Ministry of Eduction for Advanced Catalysis Materials, Department of Chemistry, Zhejiang Normal University, Jinhua 312004, China; sajidmahmood1987@zjnu.edu.cn (S.M.)

[6] Department of Physics, Zhejiang Normal University, Jinhua, Zhejiang 312004, China; ga_phy@yahoo.com (G.A.A.)

* Correspondence: zubairmkn@gcuf.edu.pk, ga_phy@yahoo.com; Tel.: (+923008923442)

Table of Contents

Figure S1: ^{1}H NMR (500 MHz, DMSO) of compound **3**. S3

Figure S2: ^{1}H NMR (500 MHz, DMSO) of compound **5a**. S3

Figure S3: ^{13}C NMR (126 MHz, DMSO) of compound **5a**. S4

Figure S4: ^{1}H NMR (500 MHz, DMSO) of compound **5b**. S4

Figure S5: ^{13}C NMR (126 MHz, DMSO) of compound **5b**. S5

Figure S6: ^{1}H NMR (500 MHz, DMSO) of compound **5c**. S5

Figure S7: ^{1}H NMR (500 MHz, DMSO) of compound **5d**. S6

Figure S8: ^{13}C NMR (126 MHz, DMSO) of compound **5d**. S6

Figure S9: ^{1}H NMR (500 MHz, DMSO) of compound **5e**. S7

Figure S10: ^{13}C NMR (126 MHz, DMSO) of compound **5e**. S7

Figure S11: ^{1}H NMR (500 MHz, DMSO) of compound **5f**. S8

Figure S12: ^{1}H NMR (500 MHz, DMSO) of compound **5g**. S8

Figure S13: ^{13}C NMR (126 MHz, DMSO) of compound **5g**. S9

Figure S14: ^{1}H NMR (500 MHz, DMSO) of compound **5h**. S9

Figure S15: ^{13}C NMR (126 MHz, DMSO) of compound **5h**. S10

Figure S16: ^{1}H NMR (500 MHz, DMSO) of compound **5i**. S10

Figure S17: MD simulation and data of compound **3**. S11

Figure S18: MD simulation and data of compound **5a**. S11

Figure S19: MD simulation and data of compound **5b**. S12

Figure S20: MD simulation and data of compound **5c**. S12

Figure S21: MD simulation and data of compound **5d**. S13

Figure S22: MD simulation and data of compound **5e**. S13

Figure S23: MD simulation and data of compound **5f**. S14

Figure S24: MD simulation and data of compound **5g**. S14

Figure S25: MD simulation and data of compound **5h**. S15

Figure S26: MD simulation and data of compound **5i**. S15

Figure S27-S28: Binding pattern of compounds. S16,S17

Figure S29: RMSD value graph of compound **3**. S18

Figure S30: RMSD value graph of compound **5c**. S18

Figure S31: RMSD value graph of compound **5g**. S19

Figure S32: RMSD value graph of compound **5h**. S19

Figure S33: RMSD value graph of compound **5i**. S20

Figure S34. Antibacterial activity of (**3**) against XDR pathogens S21

Figure S35. MIC (mg/ml) and MBC of compound (3) against XDR pathogens……………………………………………………….S21
Figure S36. MIC of **3** compound against XDR pathogens………………………………………………………………………………….S21

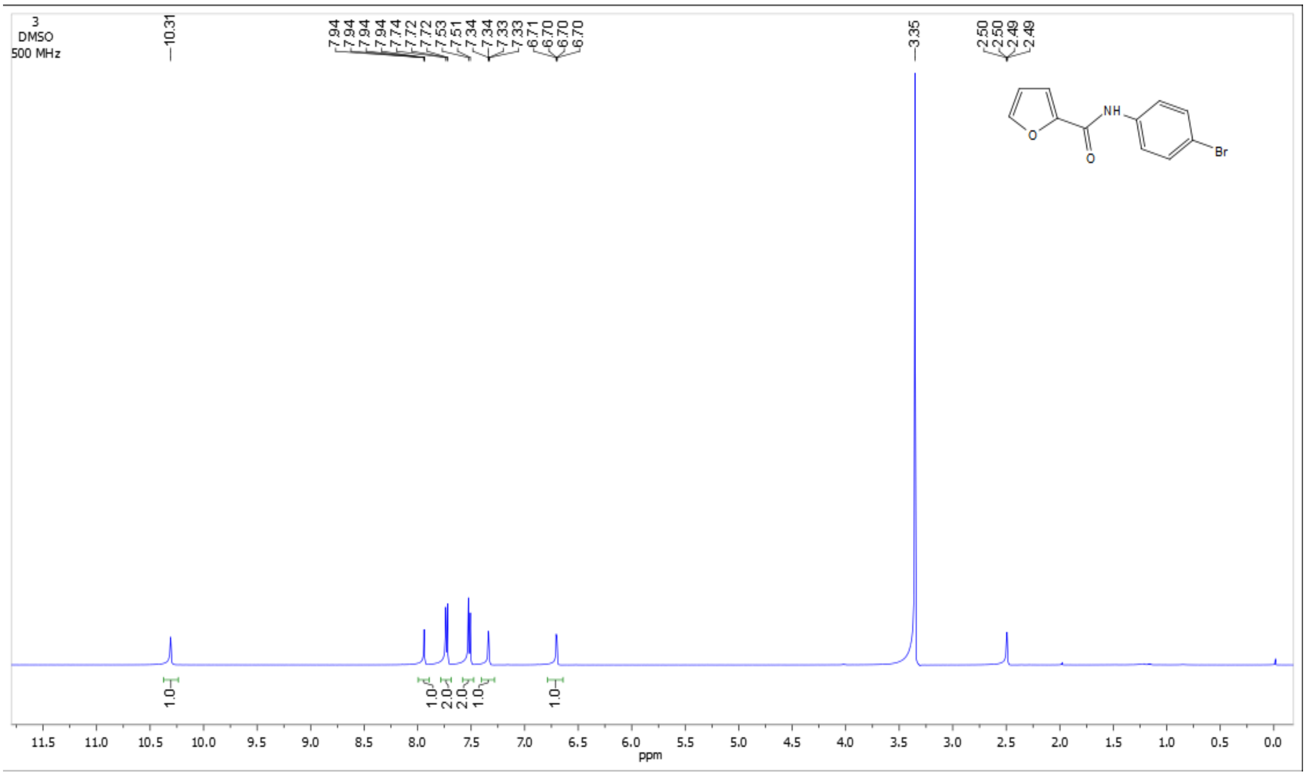

Figure S1: ^{1}HNMR (500 MHz, DMSO) of compound **3**.

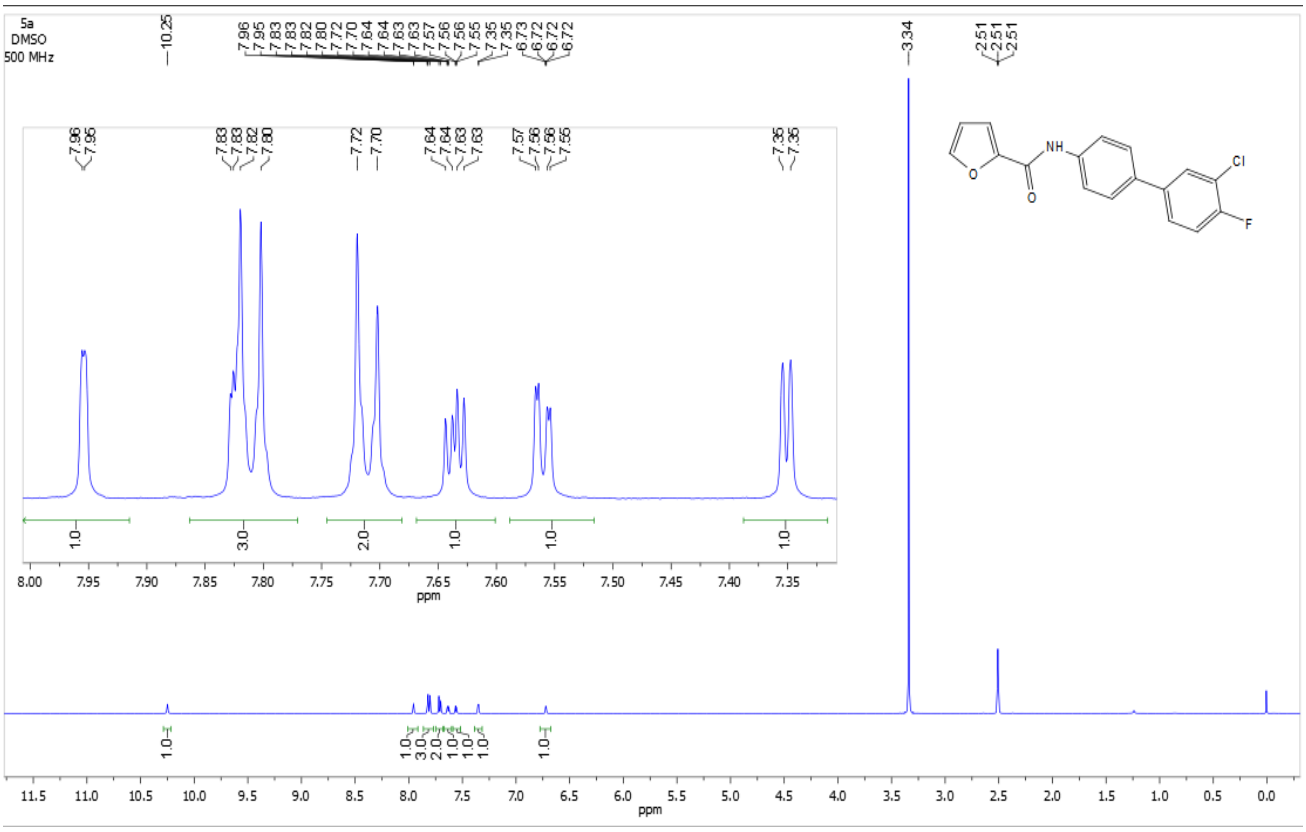

Figure S2: ^{1}HNMR (500 MHz, DMSO) of compound **5a.**

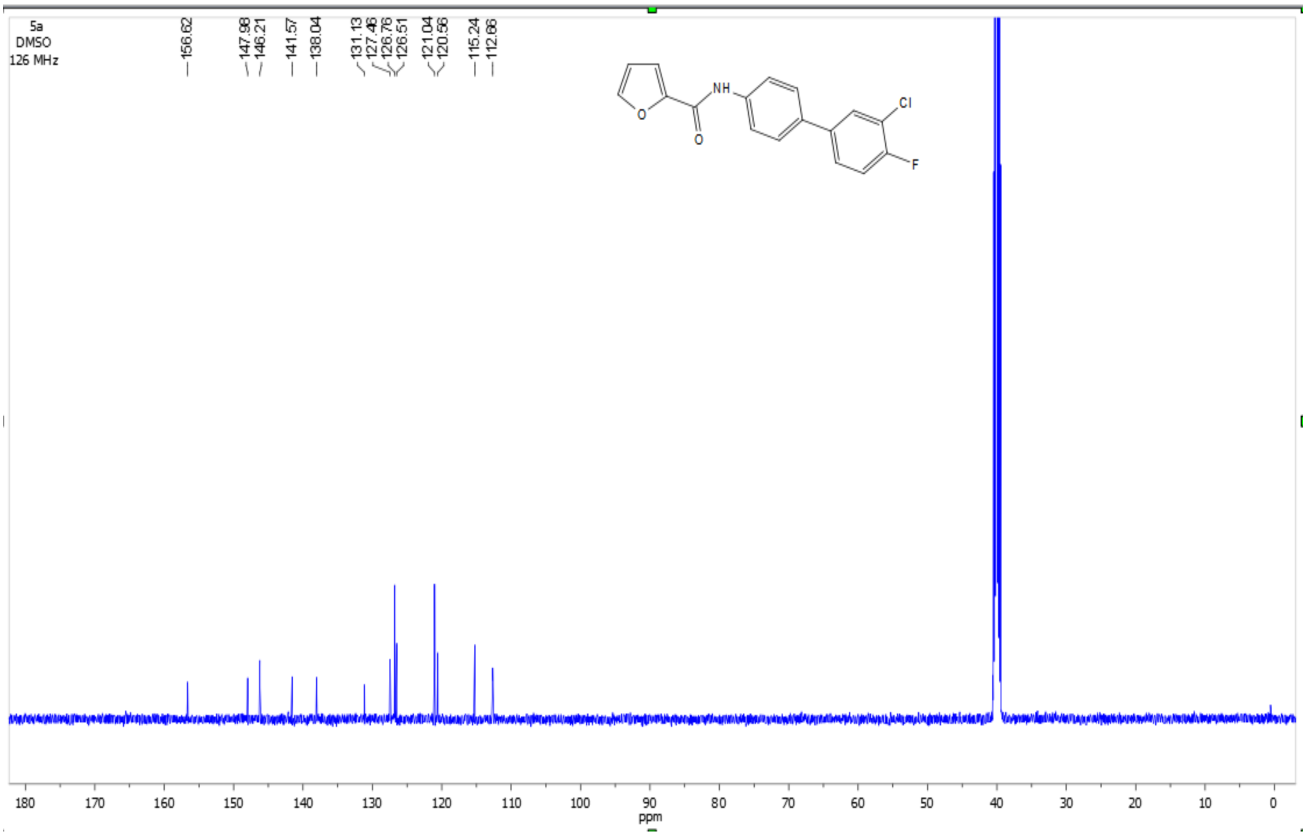

Figure S3: ^{13}CNMR (126 MHz, DMSO) of compound **5a.**

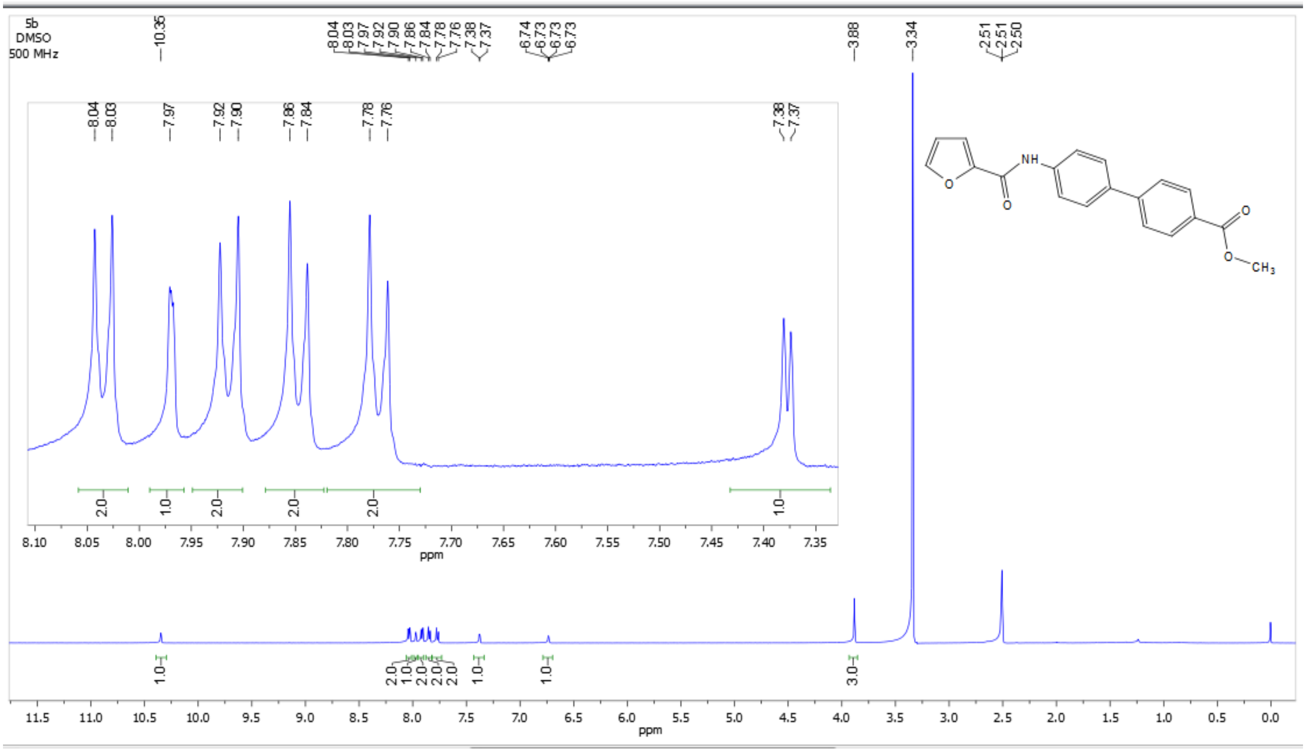

Figure S4: ^{1}HNMR (500 MHz, DMSO) of compound **5b.**

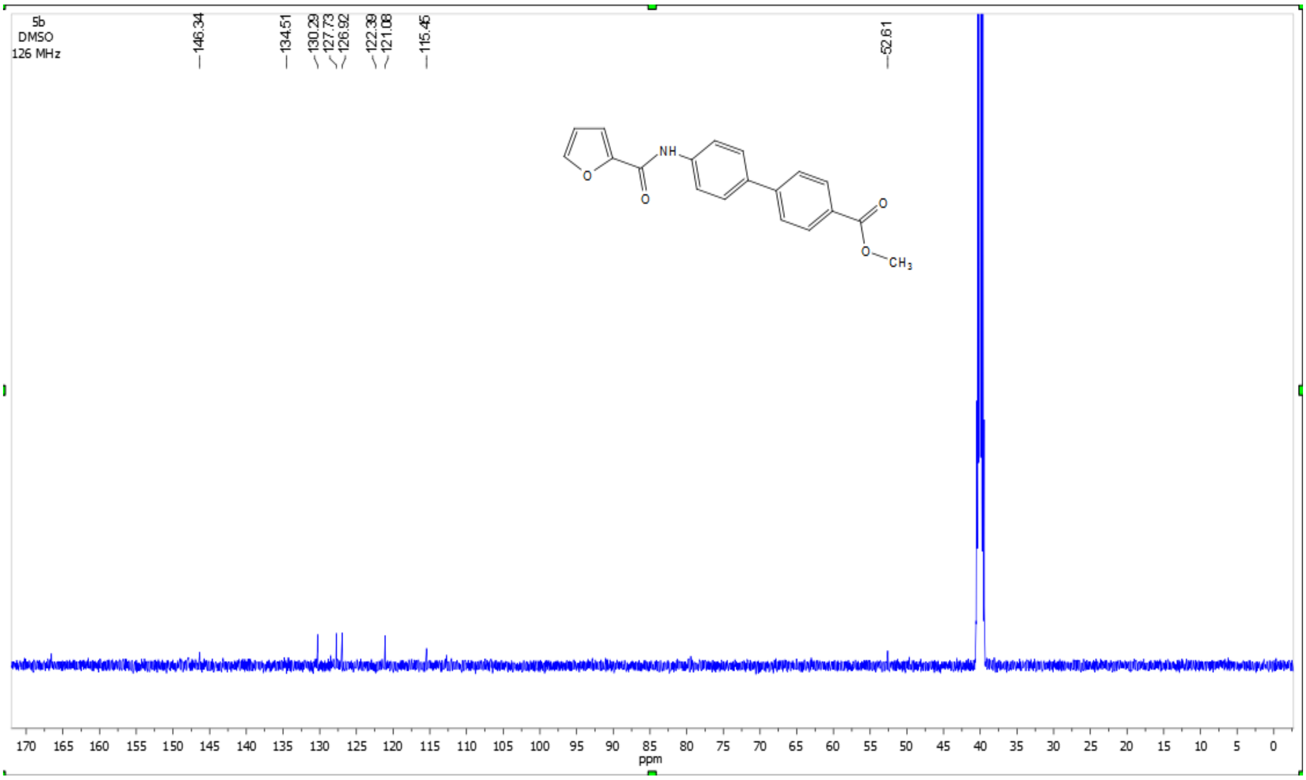

Figure S5: ^{13}CNMR (126 MHz, DMSO) of compound **5b.**

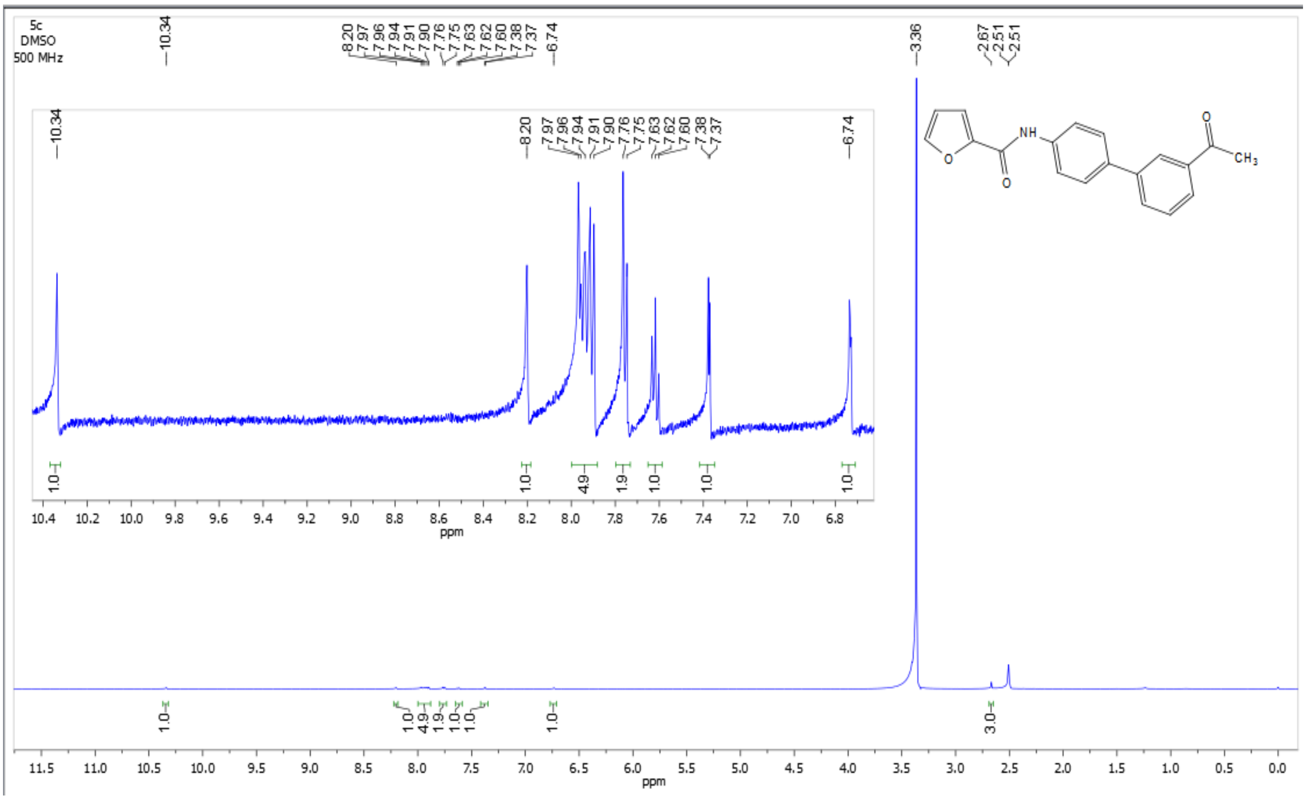

Figure S6: ^{1}HNMR (500 MHz, DMSO) of compound **5c.**

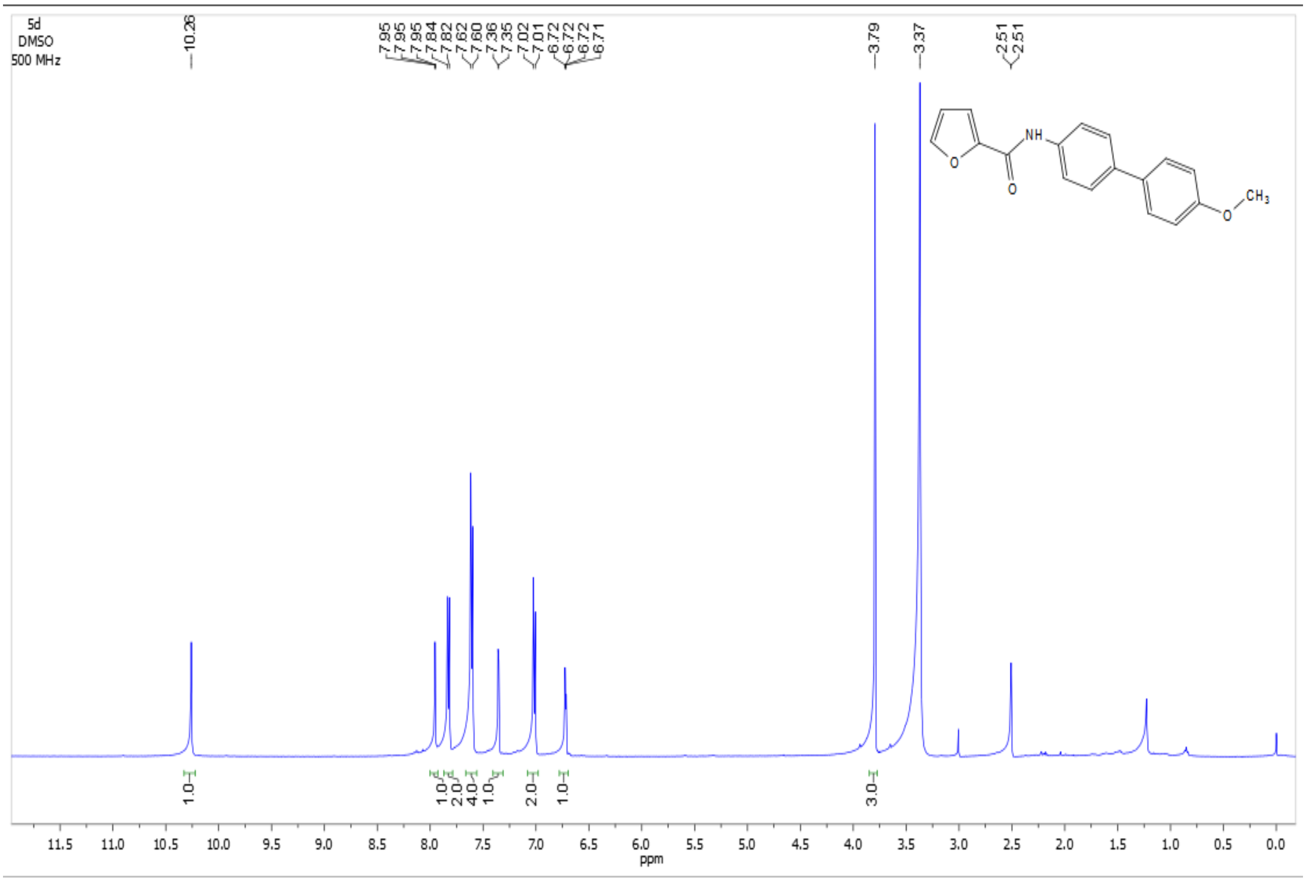

Figure S7: ^{1}HNMR (500 MHz, DMSO) of compound **5d.**

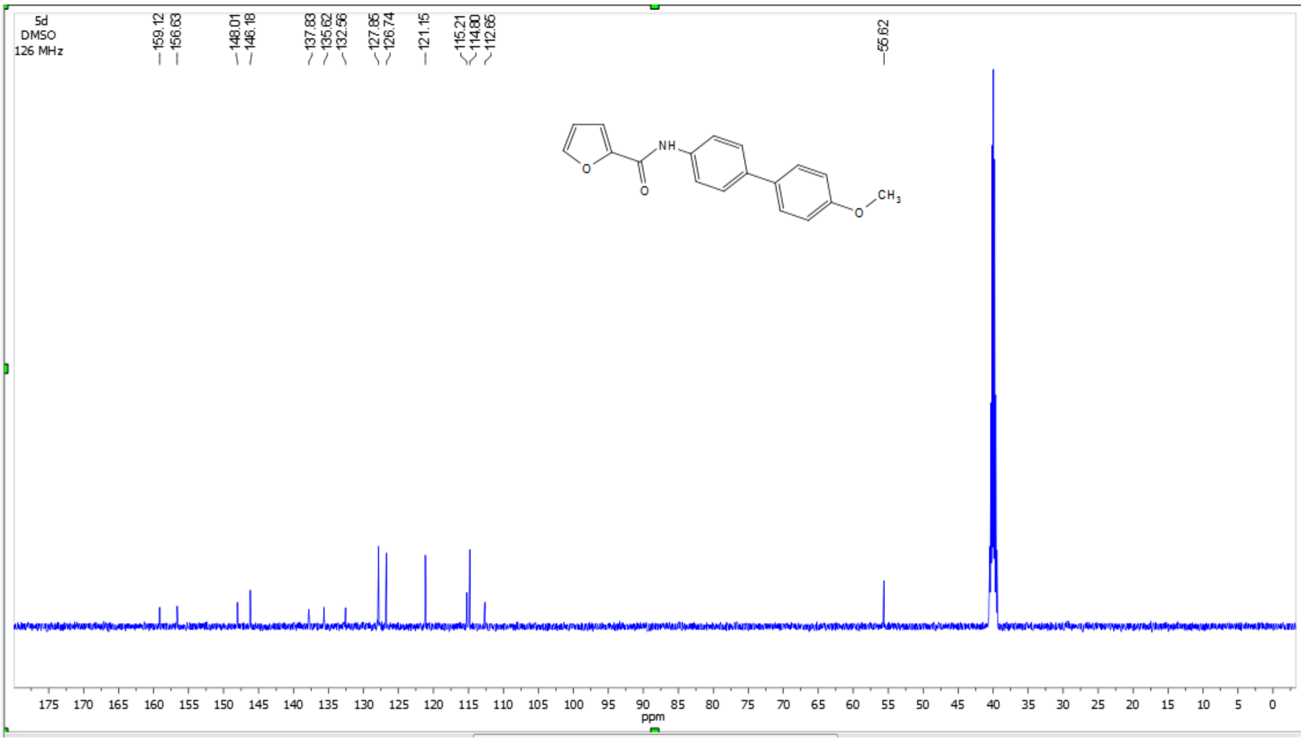

Figure S8: ^{13}CNMR (126 MHz, DMSO) of compound **5d.**

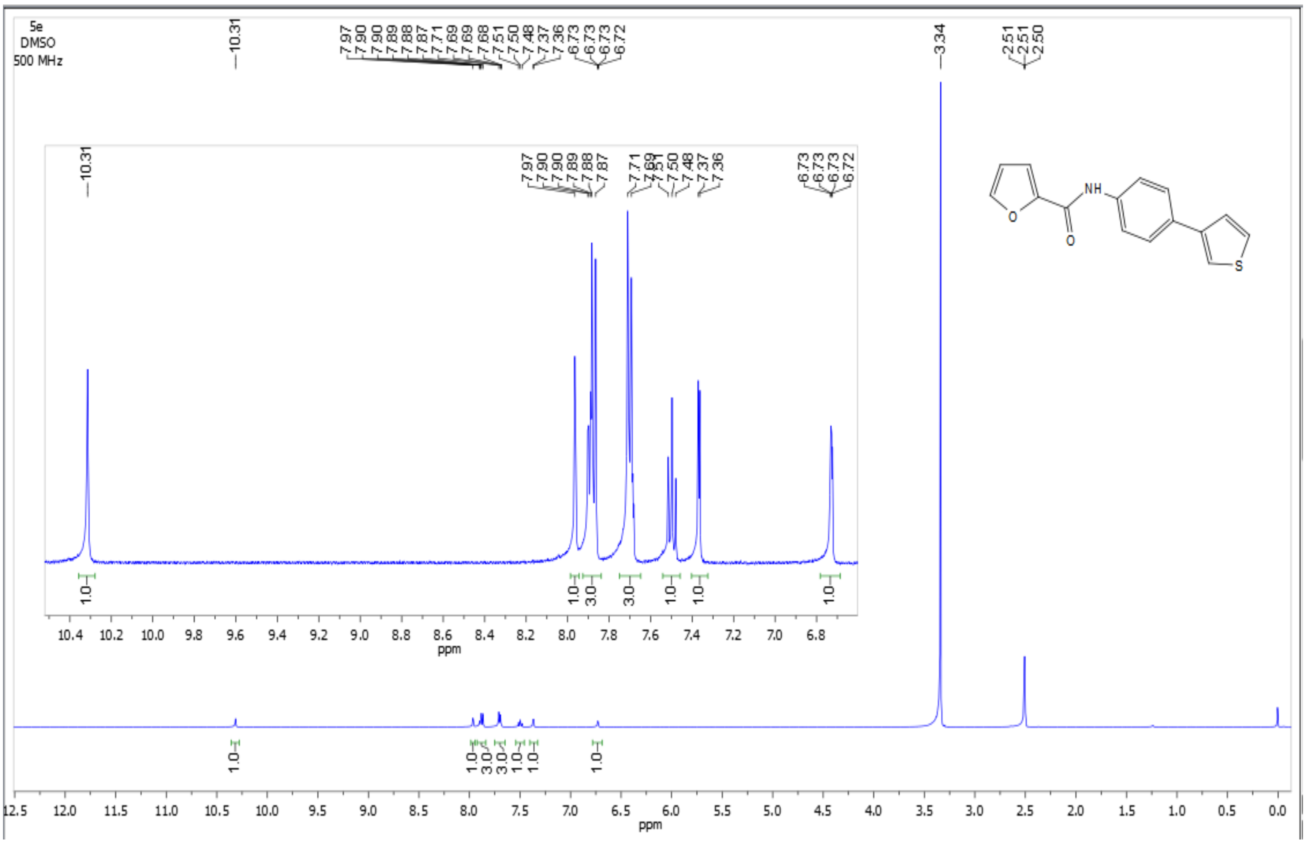

Figure S9: ^{1}HNMR (500 MHz, DMSO) of compound **5e.**

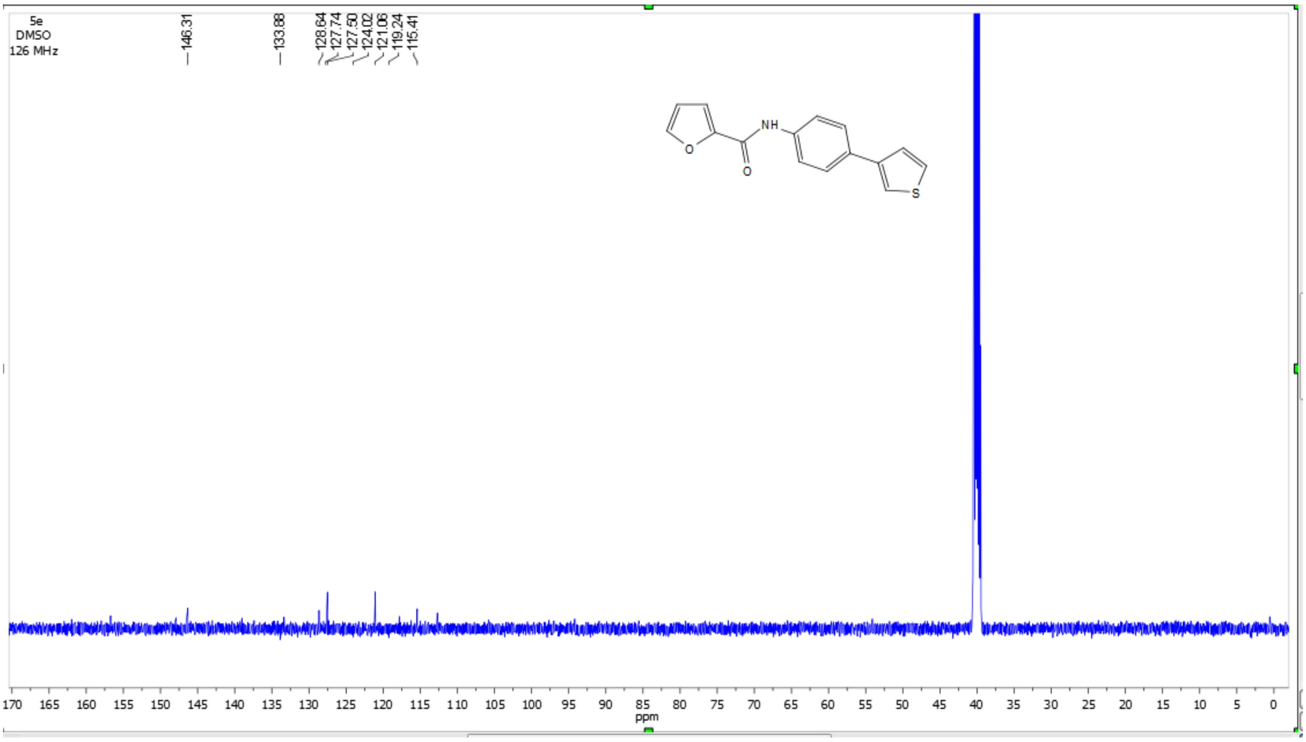

Figure S10: ^{13}CNMR (126 MHz, DMSO) of compound **5e.**

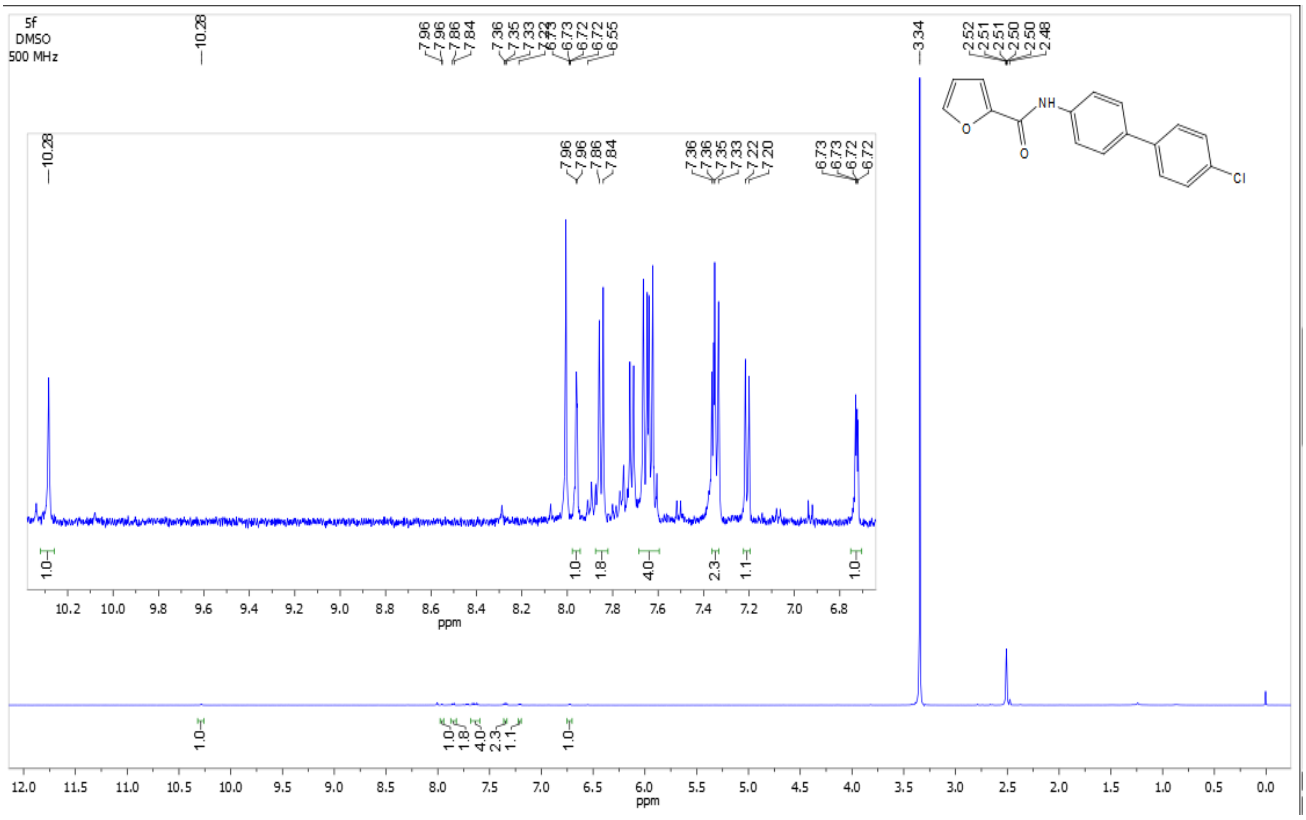

Figure S11: ^{1}HNMR (500 MHz, DMSO) of compound **5f.**

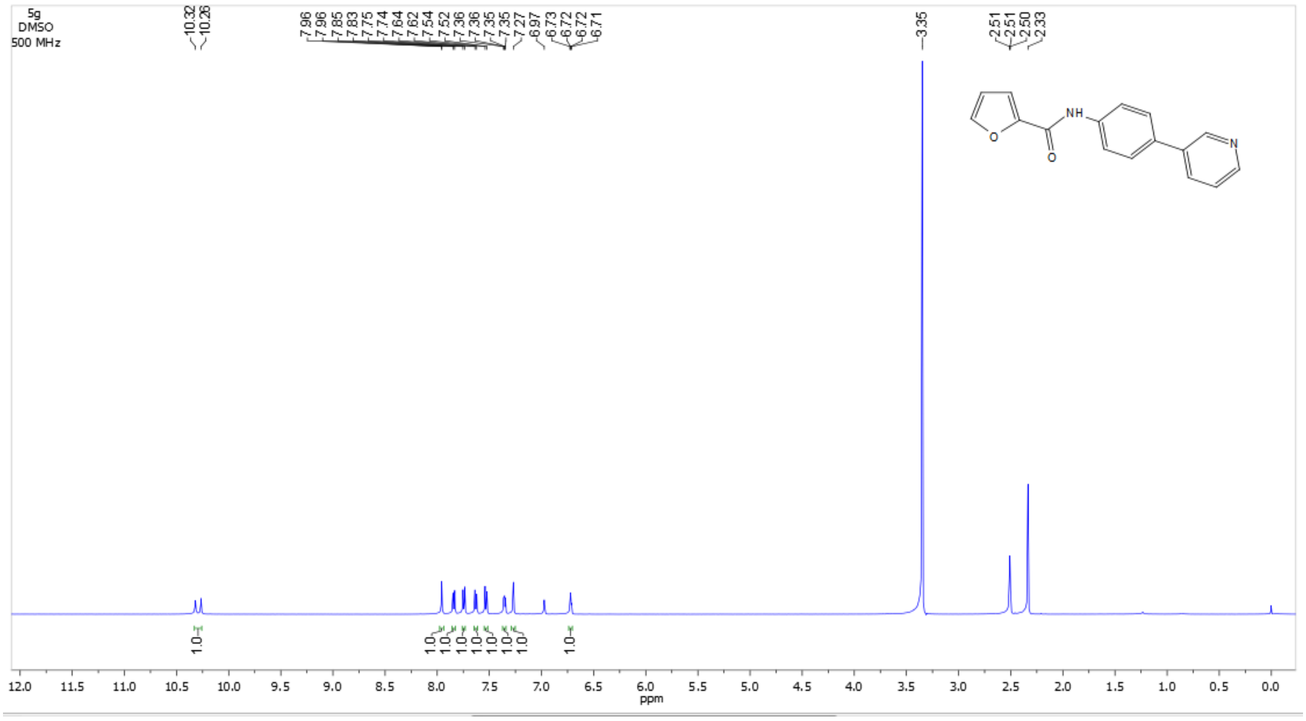

Figure S12: ^{1}HNMR (500 MHz, DMSO) of compound **5g.**

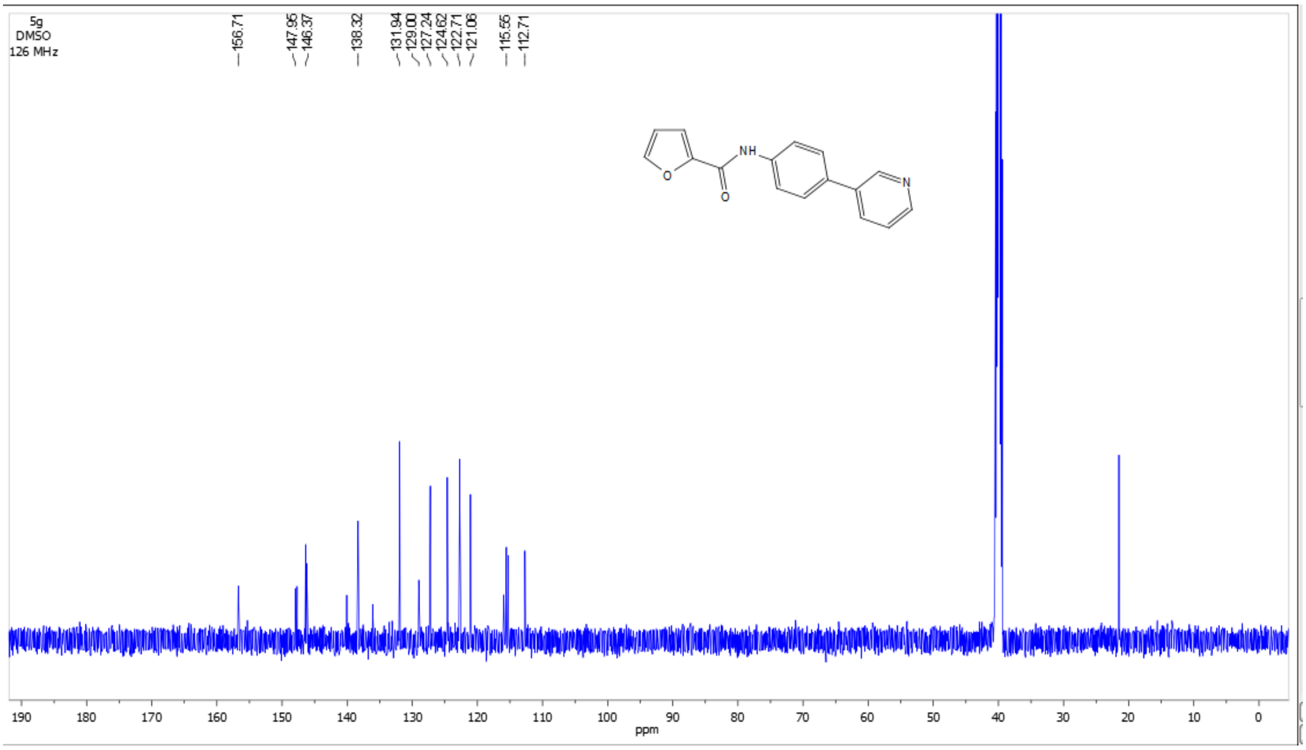

Figure S13: ^{13}CNMR (126 MHz, DMSO) of compound **5g.**

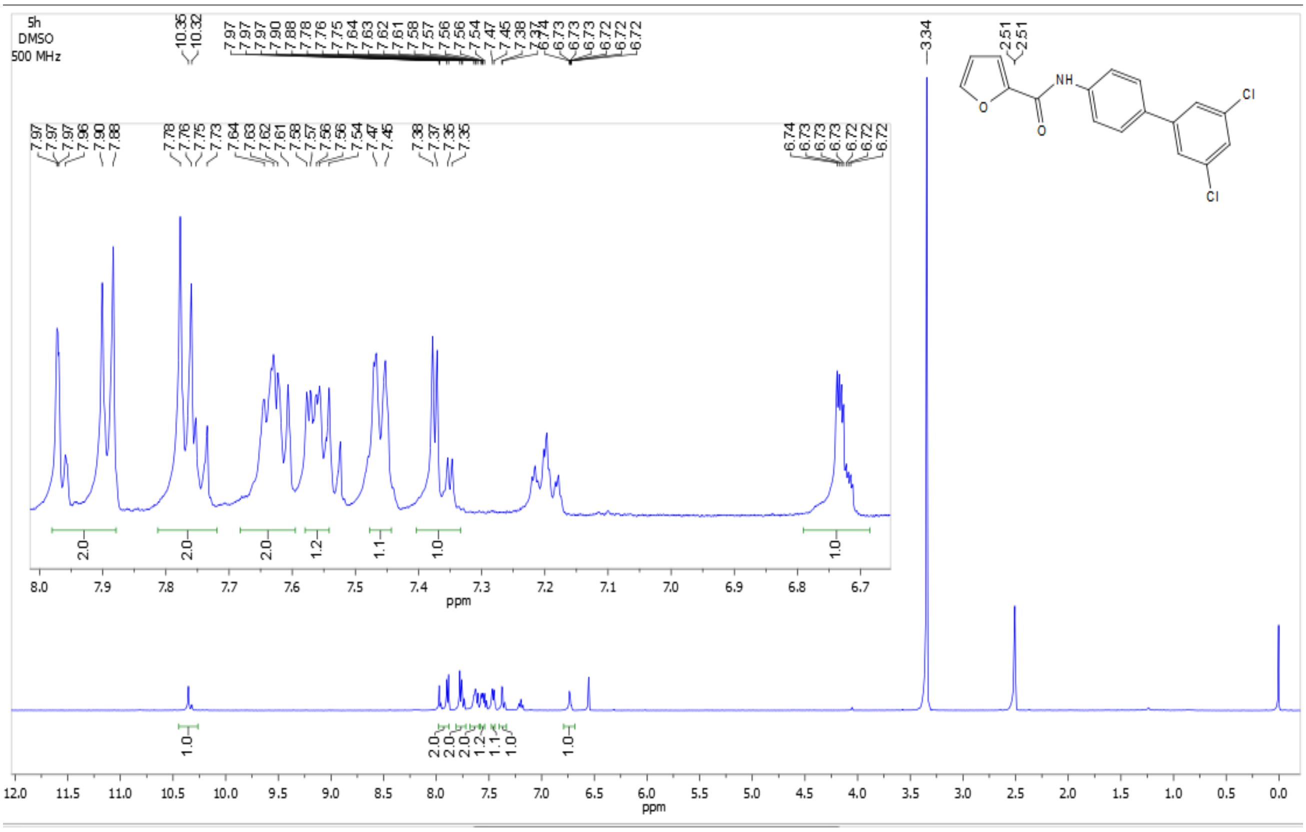

Figure S14: ^{1}HNMR (500 MHz, DMSO) of compound **5h.**

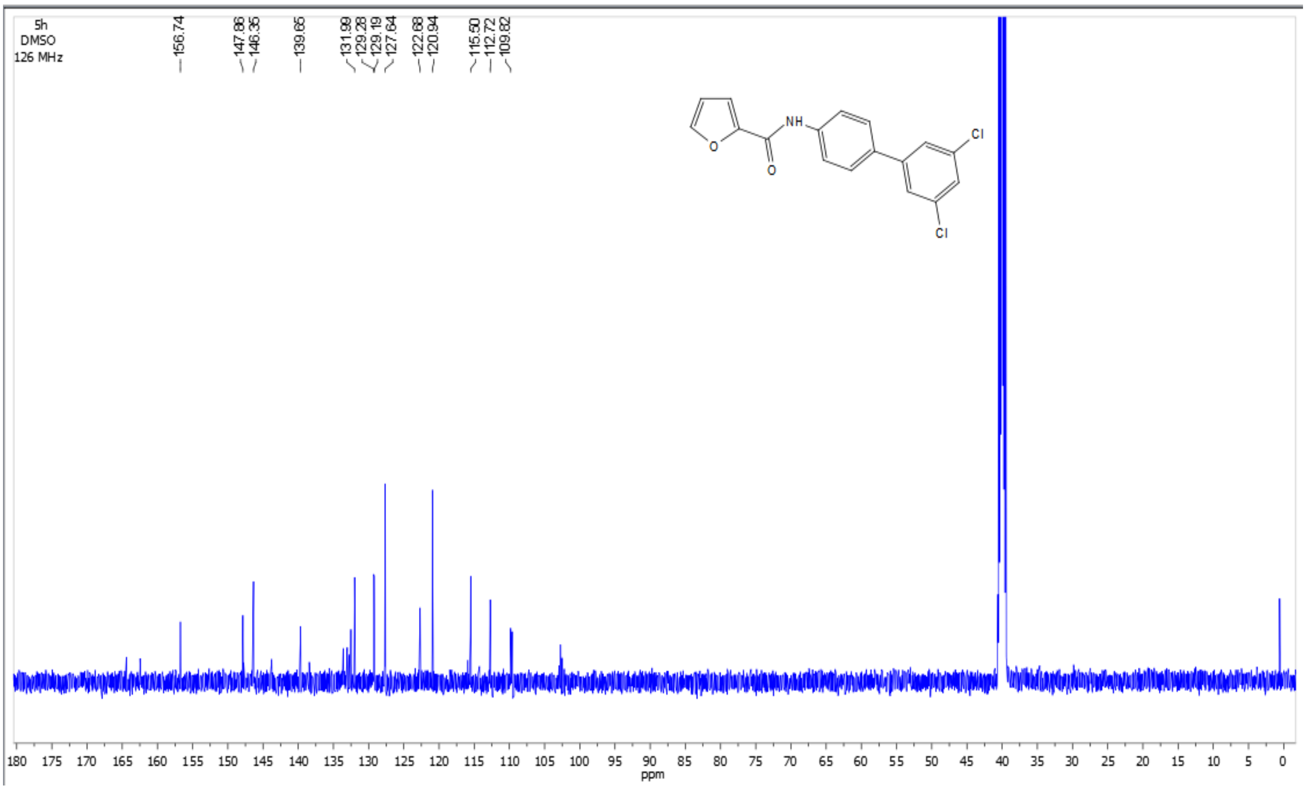

Figure S15: ^{13}CNMR (126 MHz, DMSO) of compound **5h.**

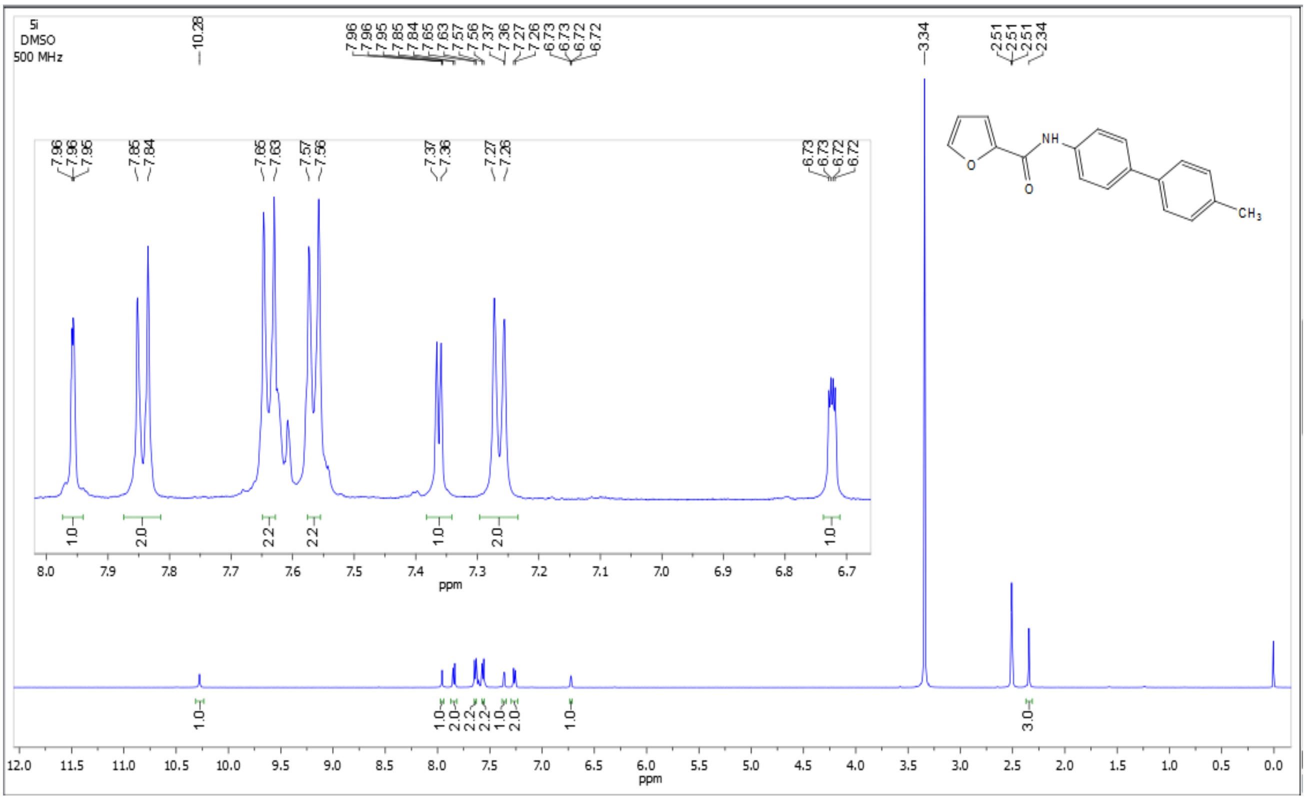

Figure S16: ^{1}HNMR (500 MHz, DMSO) of compound **5i.**

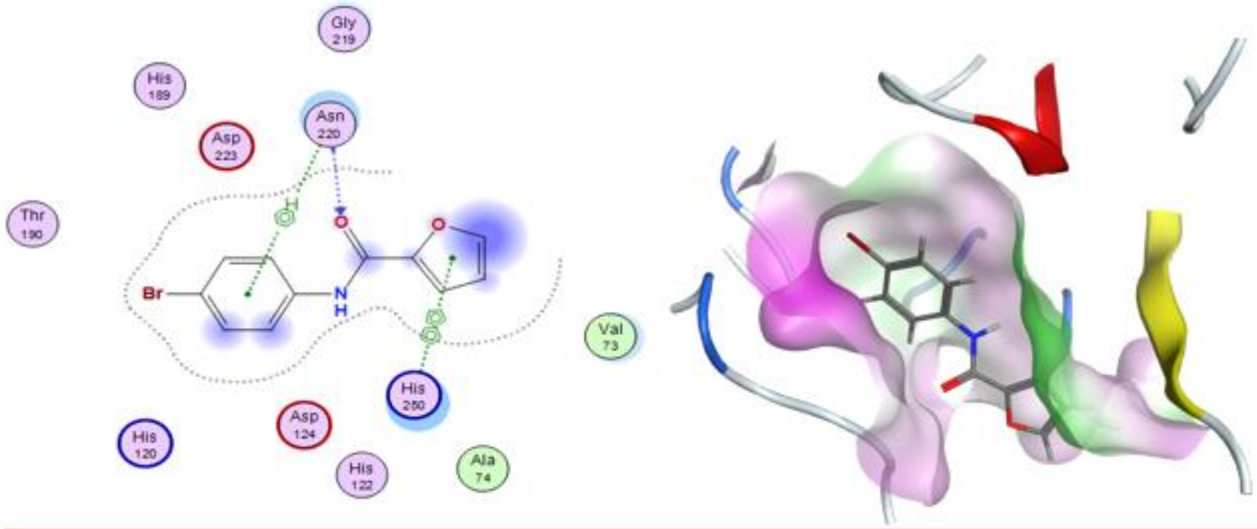

Ligand	Receptor	Interaction	Distance	Energy
O<br>6-ring<br>5-ring	N ASN 220<br>CB ASN 220<br>5-ring HIS 250	H-acceptor<br>pi-H<br>pi-pi	3.17<br>4.53<br>3.87	-0.28

Figure S17: Molecular docking and interaction pattern of compound **3**.

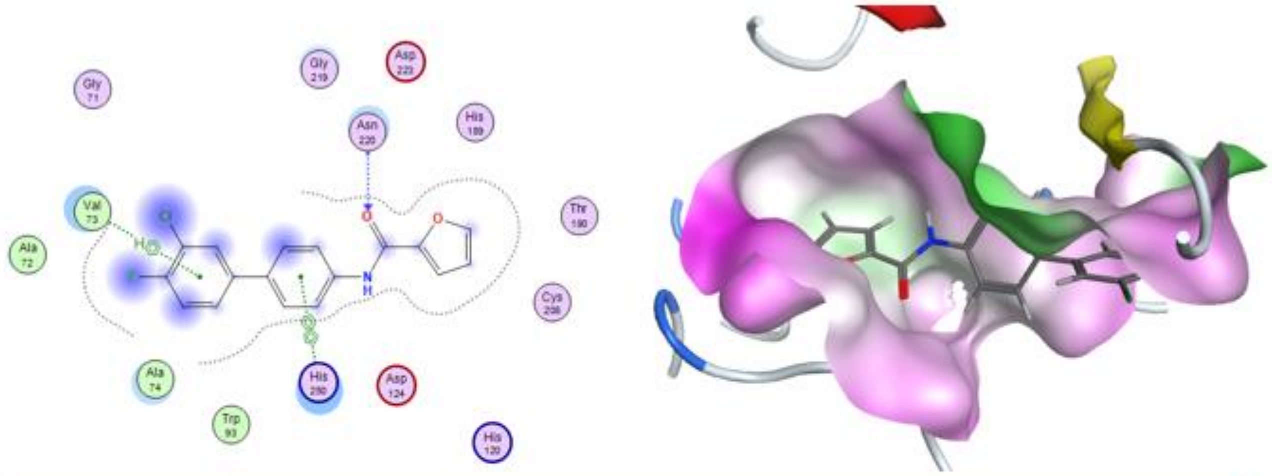

Ligand	Receptor	Interaction	Distance	Energy
O<br>6-ring<br>5-ring	N ASN 220<br>CA VAL 73<br>5-ring HIS 250	H-acceptor<br>pi-H<br>pi-pi	2.66<br>4.22<br>3.47	0.695

Figure S18: Molecular docking and interaction pattern of compound **5a**.

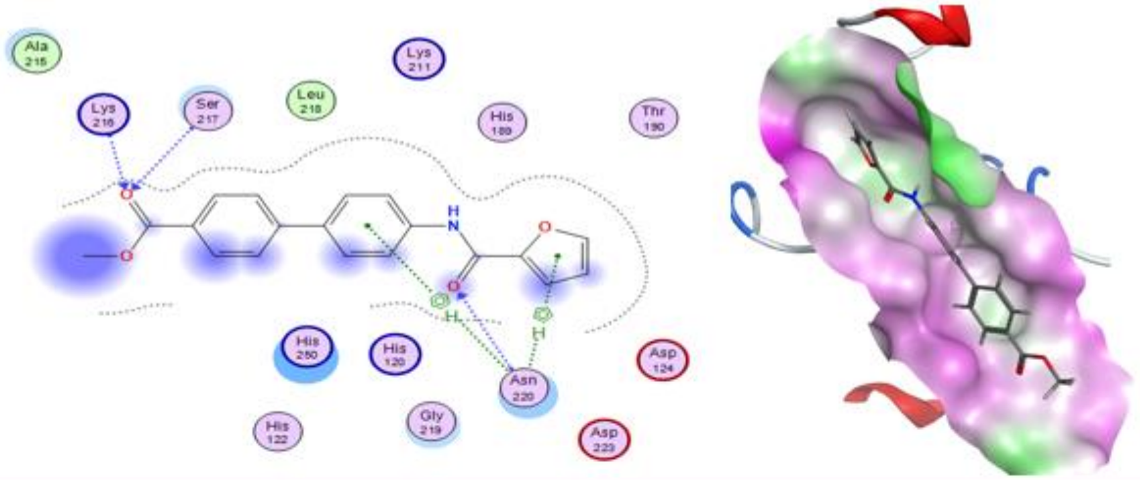

Ligand	Receptor	Interaction	Distance	Energy
O	N ASN 220	H-acceptor	3.21	-0.25
O	N LYS 216	H-acceptor	2.55	
O	N SER 217	H-acceptor	3.40	
6-ring	N ASN 220	pi-H	4.43	
5-ring	ND2 ASN 220	pi-H	3.64	

Figure S19: Molecular docking and interaction pattern of compound **5b**.

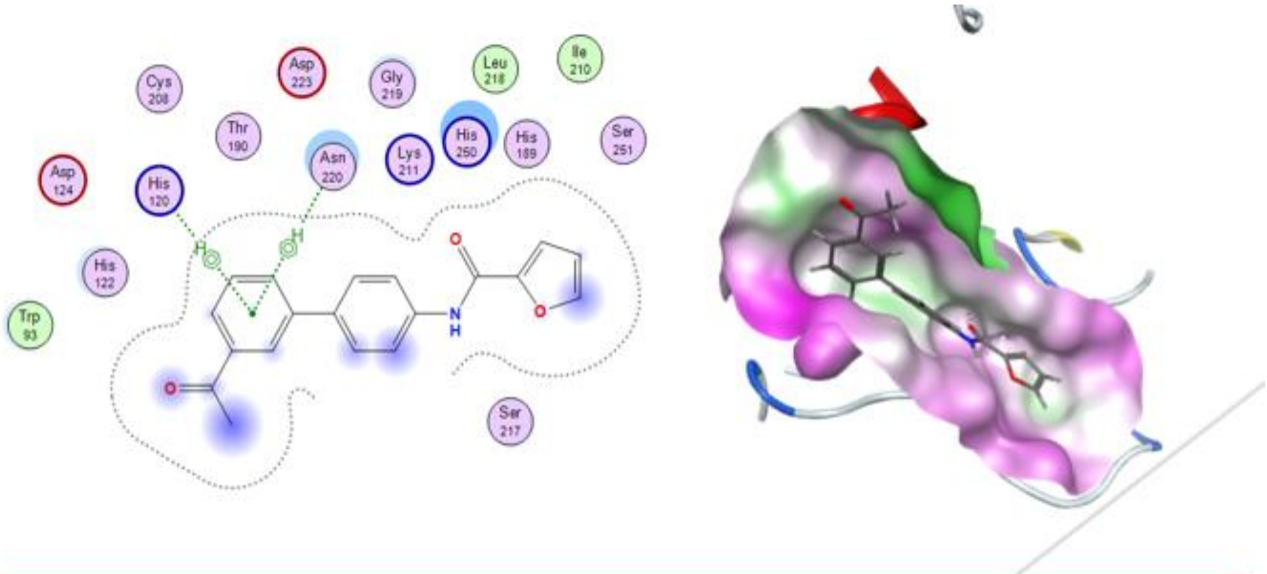

Ligand	Receptor	Interaction	Distance	Energy
O	CE1 HIS 120	pi-H	3.52	-4.5
6-ring	ND2 ASN 220	pi-H	3.67	

Figure S20: Molecular docking and interaction pattern of compound **5c**.

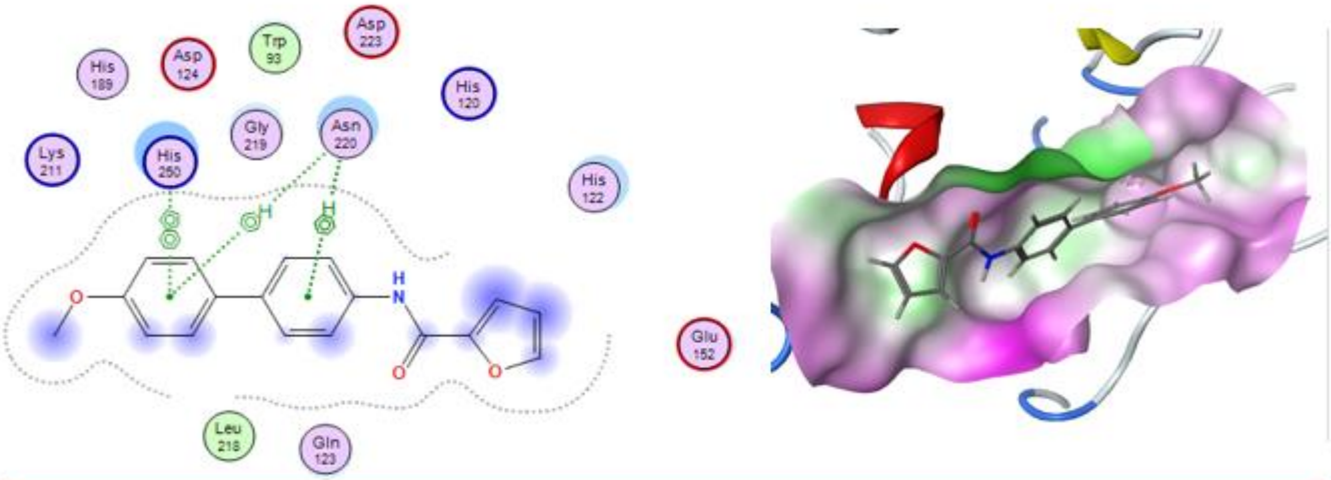

Ligand	Receptor	Interaction	Distance	Energy
6-ring	N ASN 220	pi-H	4.48	-2.1
6-ring	CB ASN 220	pi-H	4.37	
6-ring	ND2 ASN 220	pi-H	4.04	
6-ring	5-ring HIS 250	pi-pi	3.69	

Figure S21: Molecular docking and interaction pattern of compound **5d**

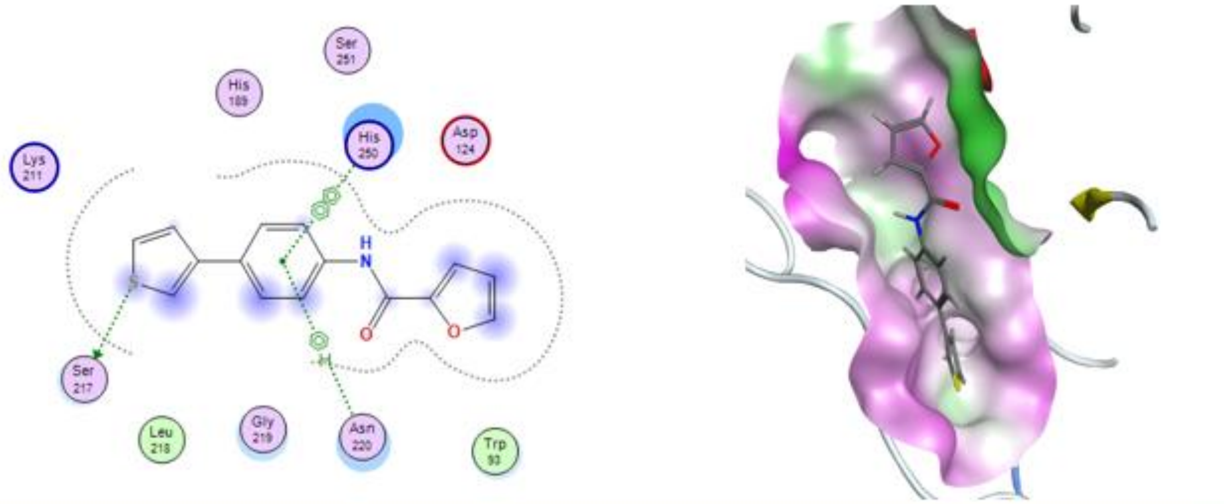

Ligand	Receptor	Interaction	Distance	Energy
S 26	OG SER 217	H-donor	3.48	-1.07
6-ring	N ASN 220	pi-H	4.32	
6-ring	5-ring HIS 250	pi-pi	3.78	
6-ring				

Figure S22: Molecular docking and interaction pattern of compound **5e**.

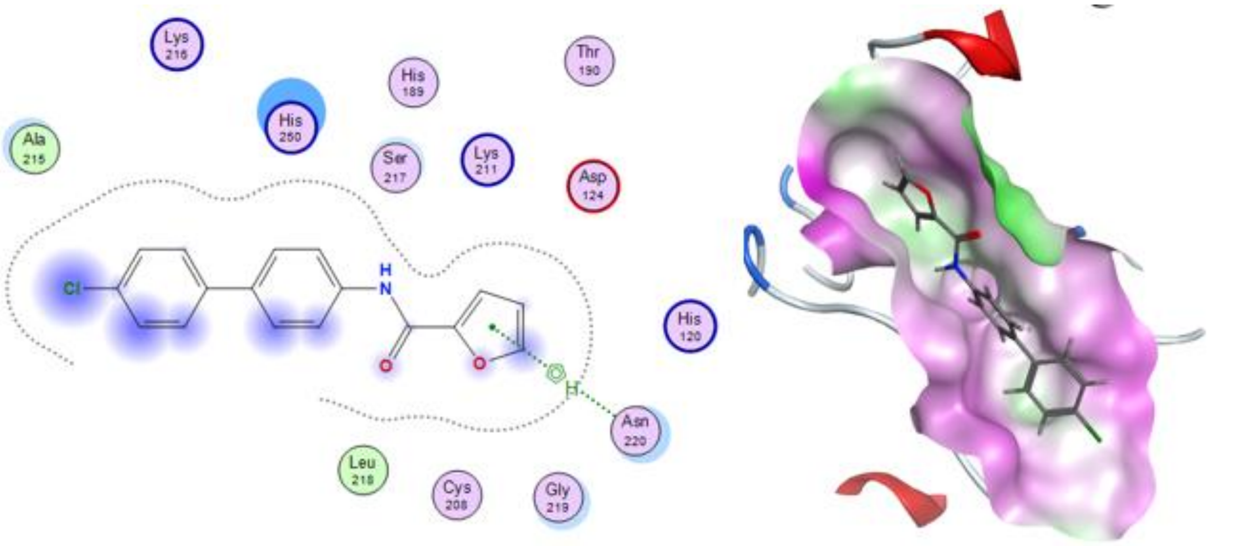

Ligand	Receptor	Interaction	Distance	Energy
6-ring	ND2 ASN 220	pi-H	4.19	

Figure S23: Molecular docking and interaction pattern of compound **5f**.

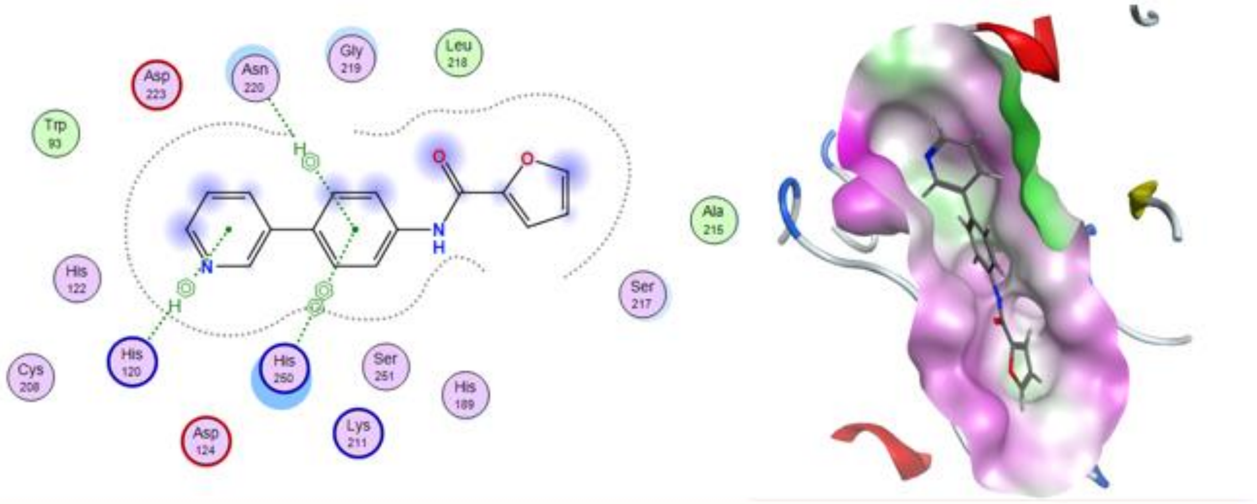

Ligand	Receptor	Interaction	Distance	Energy
6-ring	CE1 HIS 120	pi-H	4.07	-1.3
6-ring	N ASN 220	pi-H	4.29	
6-ring	5-ring HIS 250	pi-pi	3.58	

Figure S24: Molecular docking and interaction pattern of compound **5g**.

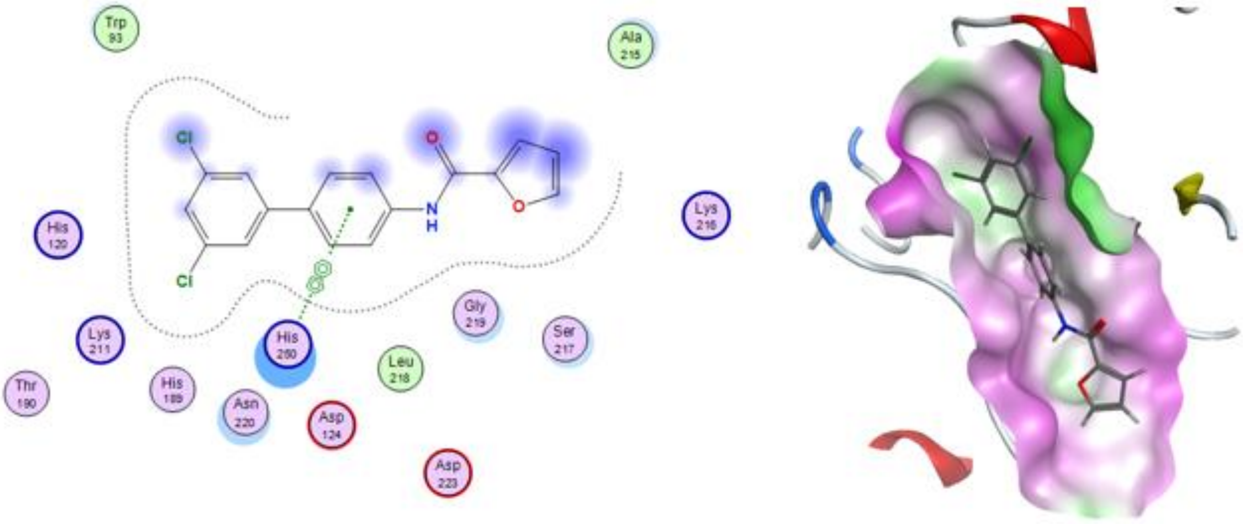

Ligand	Receptor	Interaction	Distance	Energy
6-ring	5-ring HIS 250	pi-pi	3.92	-2.17

Figure S25: Molecular docking and interaction pattern of compound **5h**.

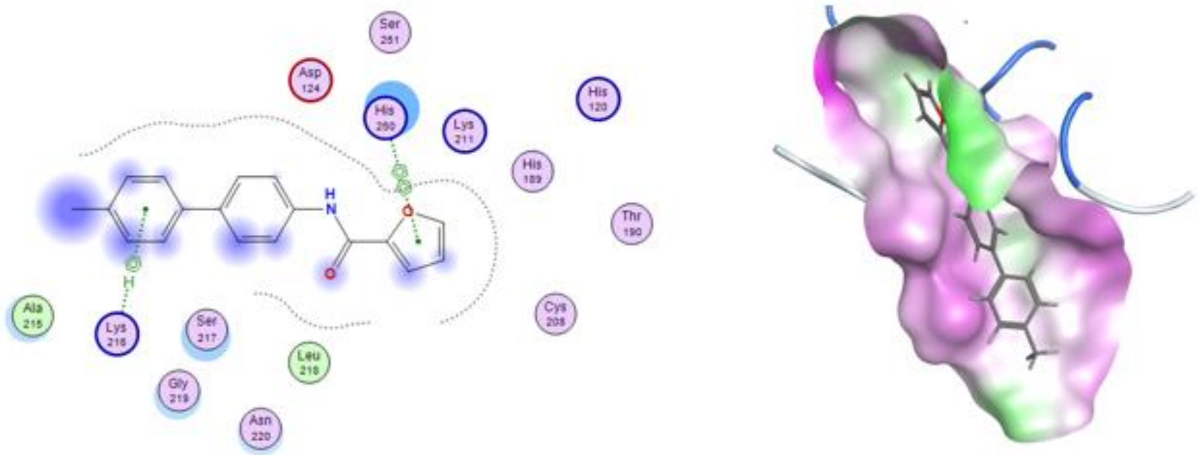

Ligand	Receptor	Interaction	Distance	Energy
6-ring	N LYS 216	pi-H	4.58	-3.78
5-ring	5-ring HIS 250	pi-pi	3.99	

Figure S26: Molecular docking and interaction pattern of compound **5i**.

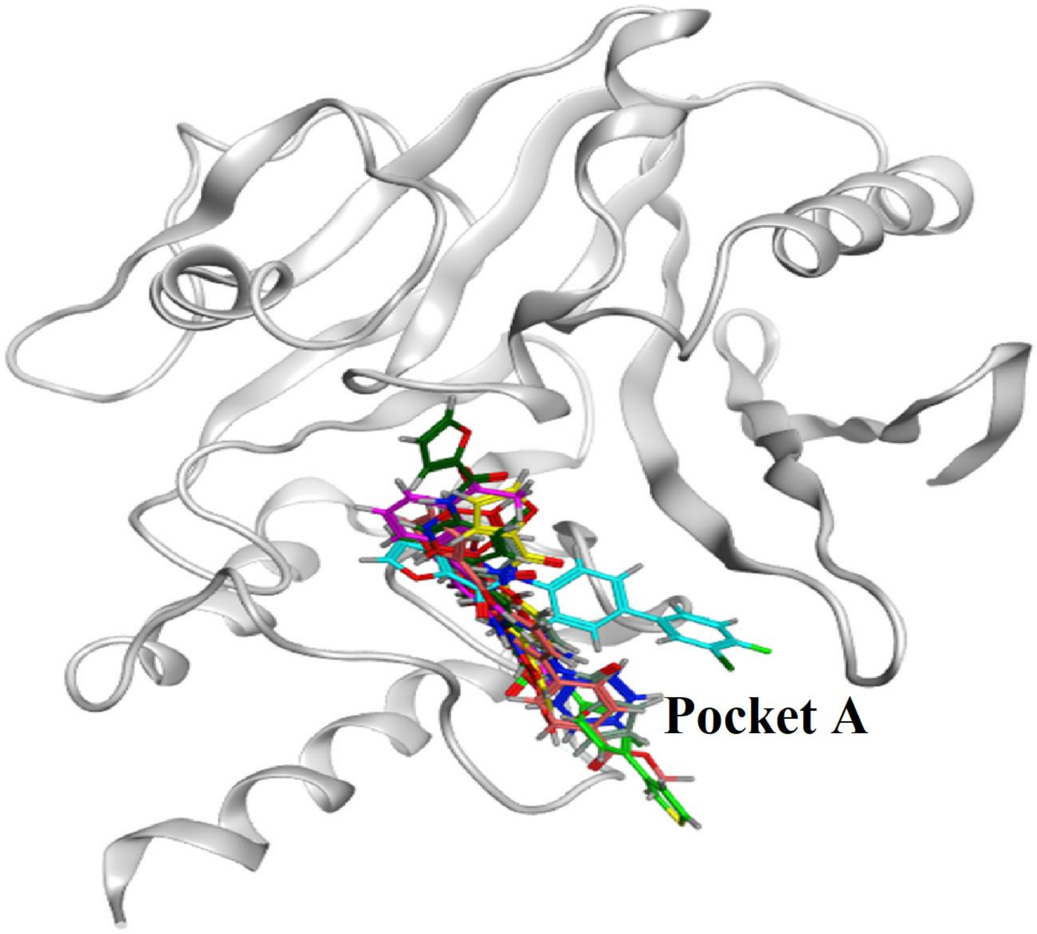

Figure S27: The selected conformation of each Ligand (3, 5a-5i) demonstrate binding in the pocket A of NDM-1 protein.

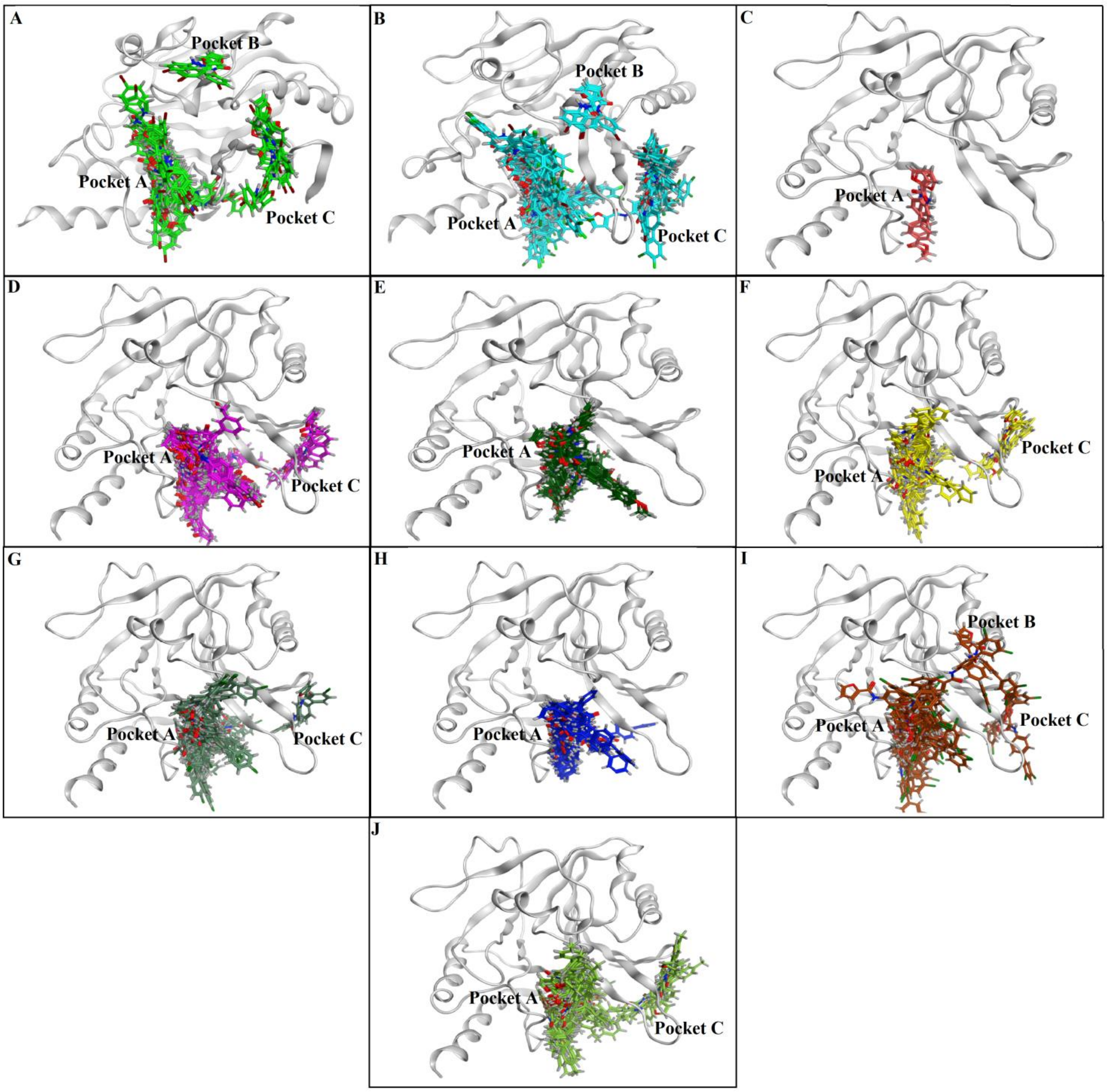

Figure S28: Ligand binding to the NDM-1 protein displaying three major binding cavities (Pocket A, Pocket B and Pocket C). It has been clearly observed that for each ligand maximum conformations are occupied in the pocket A of NDM-1 protein. (A)(C) (I) compound 3 , 5b and 5h occupies pocket A, B and C. (C)(H) compound 5b and 5g conformations only occupies pocket A. (D) (E)(F) (G)(J) Compound 5c, 5d, 5e, 5f and 5i occupies pocket A and C .

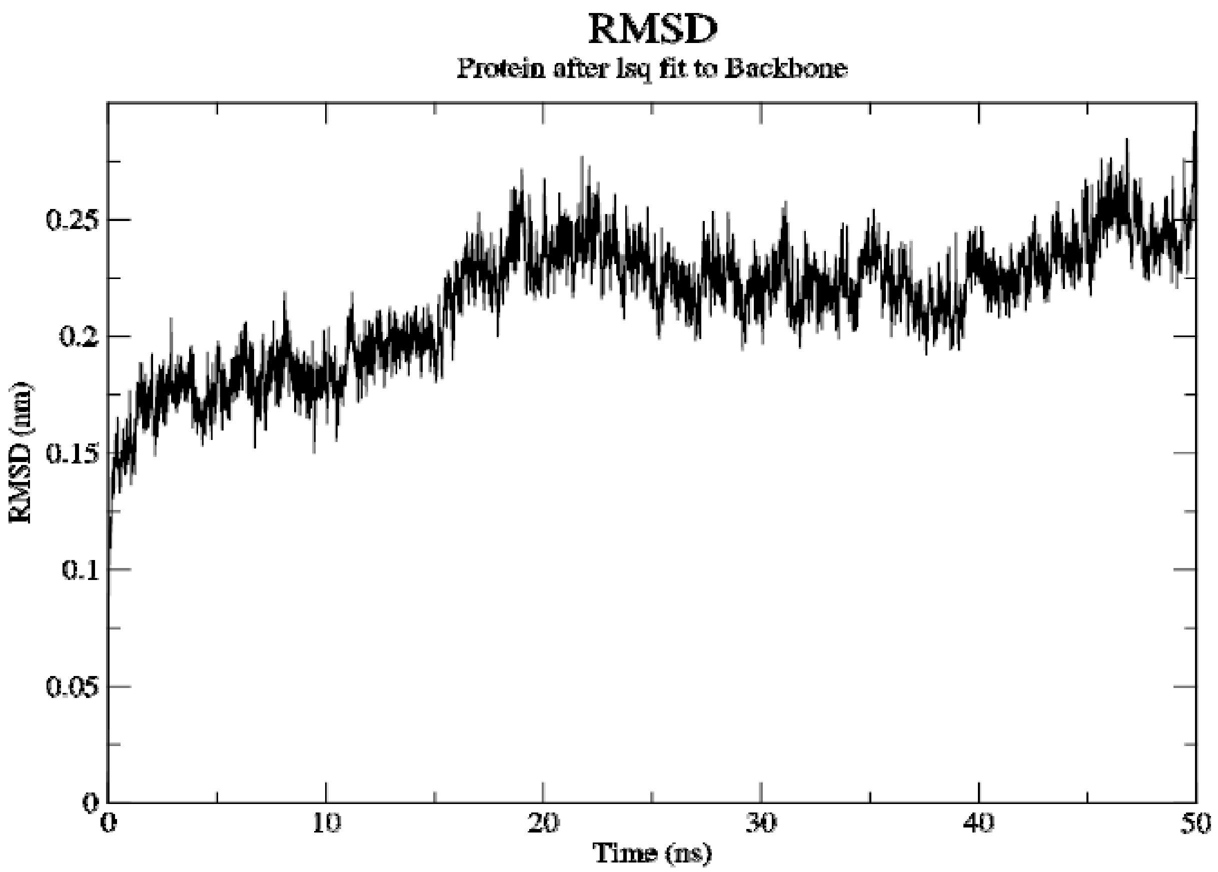

Figure S29: RMSD graph of compound **3**.

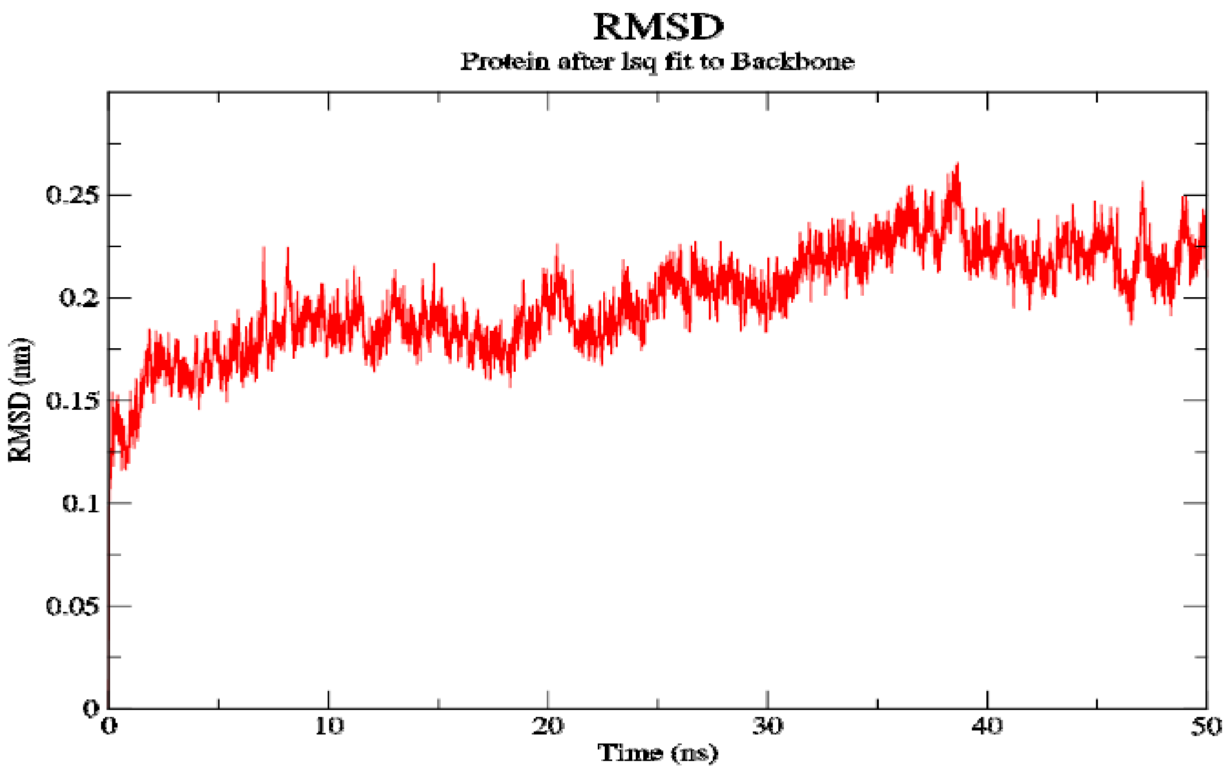

Figure S30: RMSD graph of compound **5c**.

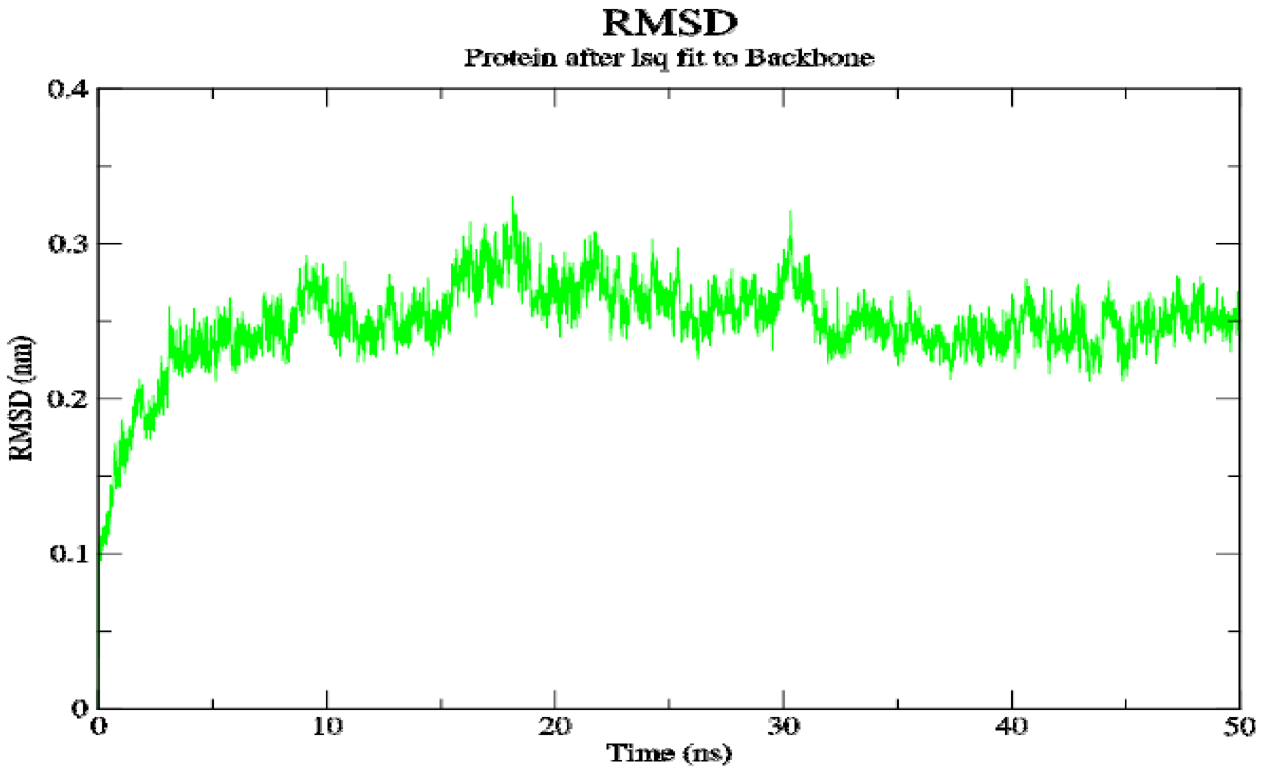

Figure S31: RMSD graph of compound **5g**.

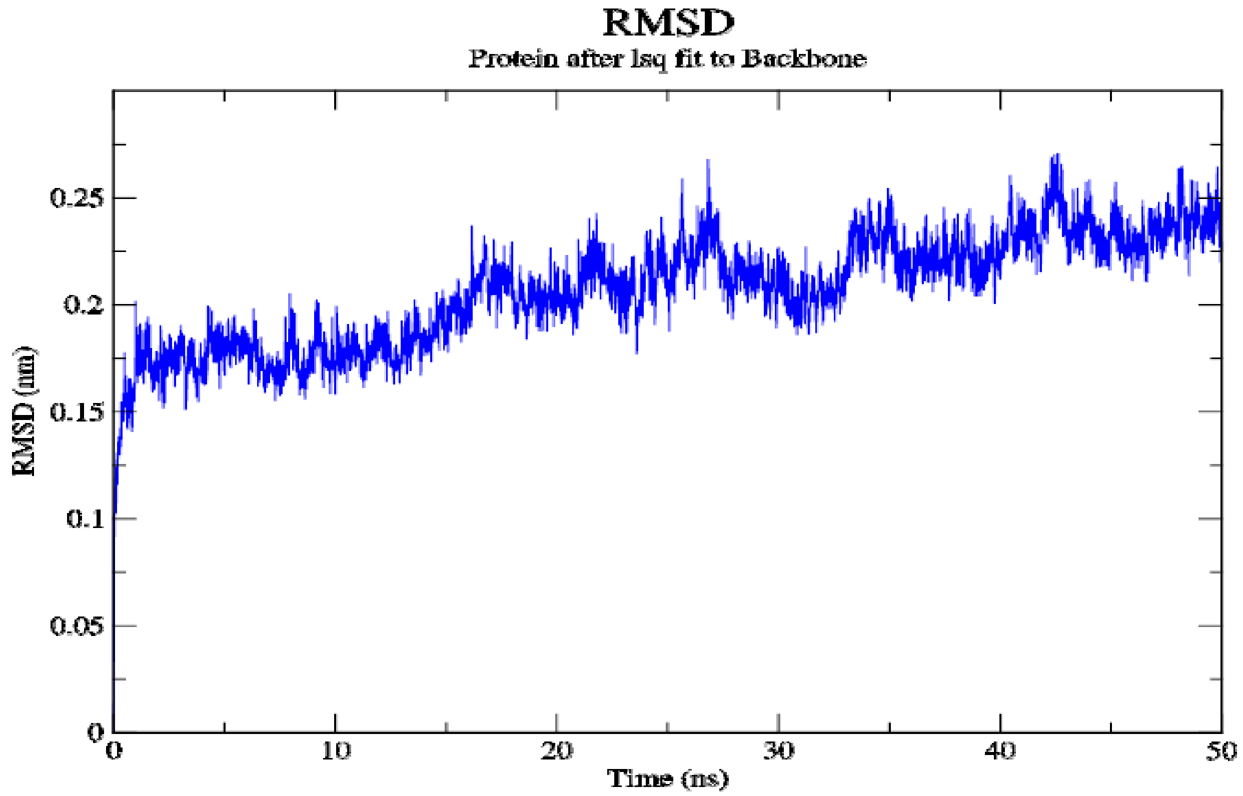

Figure S32: RMSD graph of compound **5h**.

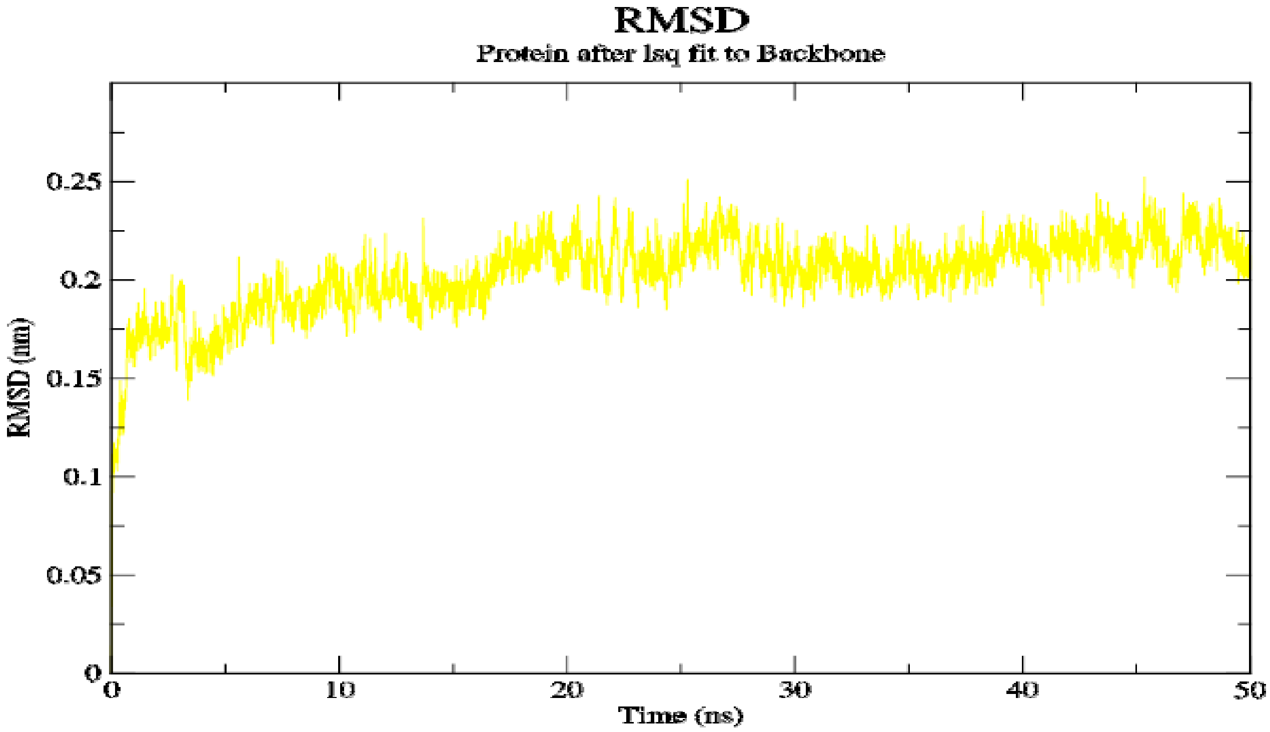

Figure S33: RMSD graph of compound **5i**.

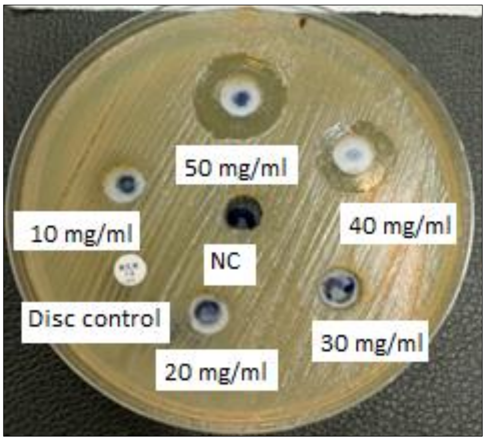

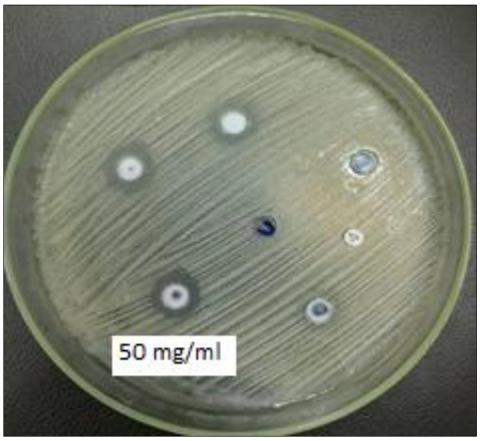

CRAB **CREC**

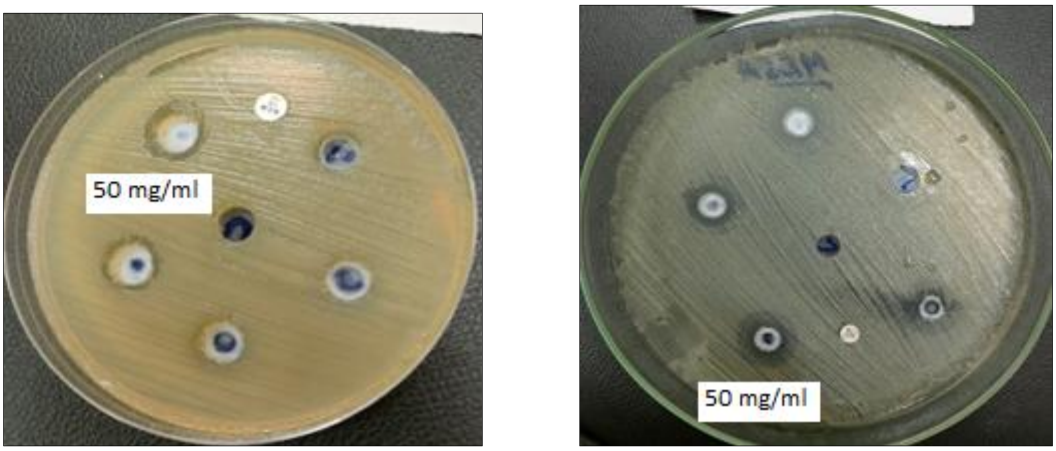

CRKP **MRSA**

Figure S34. Antibacterial activity of (**3**) against XDR pathogens.

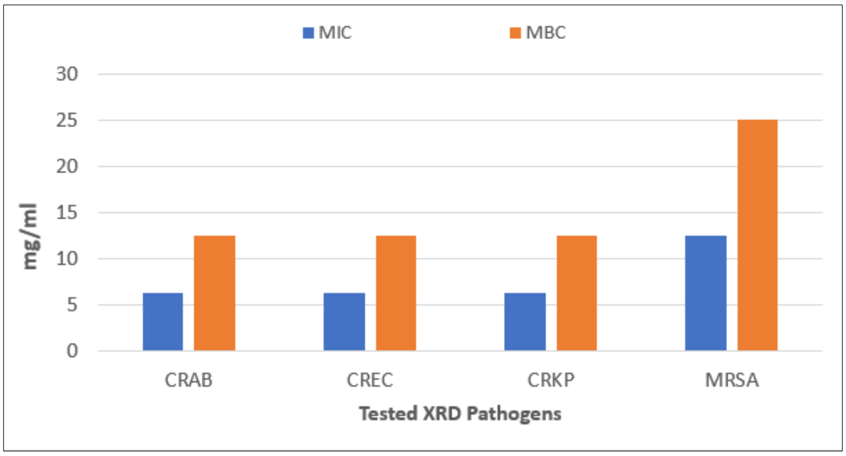

Figure S35. MIC (mg/ml) and MBC of compound **(3)** against XDR pathogens.

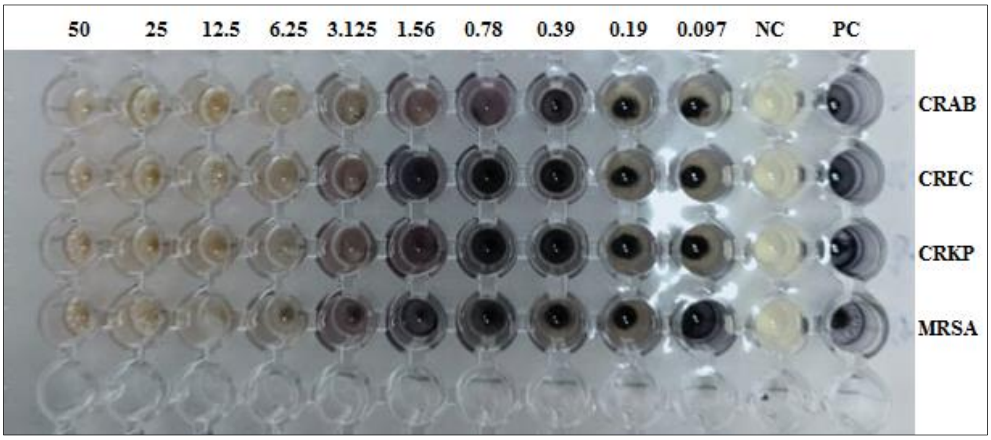

NC: Negative control; PC: Positive control

Figure S36. MIC of **3** compound against XDR pathogens
